# A new interpretation on vascular architecture of the cauline system in Commelinaceae (Commelinales)

**DOI:** 10.1371/journal.pone.0218383

**Published:** 2019-06-20

**Authors:** Ricardo SB Vita, Nanuza L. Menezes, Marco OO Pellegrini, Gladys FA Melo-de-Pinna

**Affiliations:** Department of Botany, Biosciences Institute, University of São Paulo, São Paulo, Brazil; University of Poonch Rawalakot, PAKISTAN

## Abstract

The vascular system of monocotyledons, including Commelinaceae, has been studied since the 19^th^ century, but to date, the proposed vascular architecture models consist of schematic representations partially based on the authors' interpretation. One of the greatest difficulties in studying these systems is the large number of vascular bundles and the complexity of their connections, especially in the monocotyledons which have a nodal vascular plexus. In this study, shoot apex samples of 14 species of Commelinaceae were submitted to three-dimensional analyses (confocal microscopy, X-ray microtomography, graphic vectorization, and whole-mount diaphanization), as well as conventional techniques in plant anatomy. Based on the results, a previously unreported category of bundles is described in Commelinaceae for the first time, as well as the fact that peripheral bundles are not interrupted or end blindly in the periphery of the pith, as previously thought. With this new interpretation of the vascular architecture, three patterns of nodal vascular plexus are proposed: 1) in the first pattern the internal nodal vascular plexus (IVP) forms a continuous cylinder and does not connect to the external nodal vascular plexus (EVP); 2) the IVP forms a cylinder divided into two columns and does not connect to the EVP and 3) the IVP forms a cylinder connected to the EVP. The first description of central bundles in the Commelinaceae might suggests their existence in closely related groups, such as the remaining four families of Commelinales (i.e., Haemodoraceae, Hanguanaceae, Philydraceae, and Pontederiaceae), and even in other distantly related groups of monocotyledons.

## Introduction

Monocotyledons are one of the most diverse herbaceous lineages of flowering plants, and its primary vascular system of the shoot is composed of bundles that vary in size, shape, organization and distribution, and can be continuous (i.e, sympodial bundles) or discontinuous (i.e., cauline bundle) [1–4].

For most authors, monocotyledons have three types of bundles: major (or large/larger), minor (or small/smaller) and cauline [1,2,4,5]. The major and minor bundles form leaf traces [3], while cauline bundles, are restricted to the stem or lack direct influence from the leaves [6–7].

Some species, such as *Rhapis excelsa* (Thunb.) Henry (Arecaceae), *Prionium serratum* (L. f.) Drège (Thurniaceae), *Dracaena fragrans* (L.) Ker Gawl., *Pleomele* spp., and *Cordyline* spp. (all Asparagaceae), *Strelitzia* spp. (Strelitziaceae), and *Zea mays* L. (Poaceae), have demonstrated the existence of two vascular systems based on the distribution and course of their vascular bundles: one internal and one external [3, 8]. The shoot of the monocotyledons can be further subdivided into two types based on their nodal region: 1) with a nodal vascular plexus; or 2) without a nodal vascular plexus. The first and most relevant studies on shoot vascularization in monocotyledons date back to the 19th century, with species of Arecaceae [2, 9, 10], Araceae [11], Acoraceae [11], Poaceae [6], Commelinaceae [6, 12] and Velloziaceae [7]. In these studies, the authors present schemes of vascular architecture, indicating the existence of different groups of vascular bundles based on their origin and distribution, as well as a proposed nomenclature and suggested course for each type of bundle. These schemes are based on the authors interpretation, and not a direct representation of the analyzed structure [3]. These works have proposed different and sometimes new nomenclatures for the vascular bundles, as well as, to their course and distribution. This has caused a great deal of confusion, since it has allowed scholars find more than one name for the same kind of bundle or more than one type of bundle with the same name.

The elevated complexity of vascularization in monocotyledons, which is due to the large number of vascular bundles in their shoot, has made it difficult to understand their vascular architecture [4], especially for researchers who are not directly involved in their study [8]. This complexity, added to the presence of a nodal vascular plexus, is the reason why this subject has long been avoided by botanists [13]. In the region of the nodal vascular plexus there is a proliferation of tracheary elements interconnecting vascular bundles of the stem to leaf traces and cauline bundles, forming an anastomosed network of vascular elements. These tracheary elements are usually tracheids, predominant in lateral connections between adjacent bundles [2, 7], which establish connections in all directions [14].

Although previous studies show the existence of a nodal vascular plexus in Commelinaceae, little information is available about its structure. Considering the variation of vascular system between stem and leaves in monocotyledons, we selected 14 species of Commelinaceae for the present survey. Theses species served as models to investigate the patterns of vascular architecture in the family, based on complementary three-dimensional analyses, such as confocal microscopy, X-ray microtomography (μCT), graphic vectorization and whole-mount diaphanization.

## Material and methods

### Analyzed specimens

For the present study, 14 herbaceous species belonging to subfamily Commelinoideae and representing tribes Commelineae and Tradescantieae (subtribes Dichorisandrinae and Tradescantiinae), were selected (Table 1). In this study were analysed specimens of *Aneilema beninense* (P. Beauv.) Kunth (S1A Fig), *Callisia repens* (S1B Fig), *Commelina benghalensis* L. (S1C Fig), *Commelina rufipes* var. *glabrata* (D.R.Hunt) Faden & D.R.Hunt (S1D Fig), *Dichorisandra radicalis* Nees & Mart. (S1E Fig), *D*. *thyrsiflora* J.C.Mikan (S1F Fig), *Floscopa glabrata* (Kunth) Hassk. (S2A Fig), *Floscopa* aff. *glabrata* (S2B Fig), *Tradescantia cerinthoides* (S2C Fig), *Tradescantia fluminensis* (S2D Fig), *Tradescantia pallida* (Rose) D.R.Hunt (S2E Fig), *Tradescantia zebrina* (S2F Fig), *Tripogandra diuretica* (Mart.) Handlos (S3A Fig), *and Tripogandra warmingiana* (Seub.) Handlos (S3B Fig). All species analyzed are perennial herbs, with the majority being terrestrial, except for the aquatic *Floscopa* Lour., for the facultatively rupicolous and epiphytic *Callisia repens* (Jacq.) L. and *Tradescantia cerinthoides* Kunth, the facultavively epiphytic *Tradescantia fluminensis* Vell. and *Tradescantia zebrina* Heynh. *ex* Bosse, and the facultatively aquatic *Tripogandra diuretica* (Mart.) Handlos.

**Table 1:** List of studied species from the family Commelinaceae, with respective sample voucher and collection locality.

Species	Voucher	Collection Locality
Subfamily Commelinoideae		
Tribe Commelineae		
*Aneilema beniniense* (P. Beauv.) Kunth	Pellegrini 218 (RB)	cult. Jardim Botânico do Rio de Janeiro (Brazil)
*Commelina benghalensis* L.	HUESC 15738	Ilhéus, Bahia (Brazil)
*Commelina rufipes* var. *glabrata* (D.R.Hunt) Faden & D.R.Hunt	HUESC 15742	Ilhéus, Bahia (Brazil)
*Floscopa glabrata* (Kunth) Hassk.	Pellegrini 453 (RB)	Rio de Contas, Bahia (Brazil)
*Floscopa* aff. *glabrata*	Pellegrini 466 (RB)	Santa Teresa, Espírito Santo (Brazil)
Tribe Tradescantieae		
Subtribe Dichorisandrinae		
*Dichorisandra radicalis* Nees & Mart.	HUESC 15733	Ilhéus, Bahia (Brazil).
*Dichorisandra thyrsiflora* J.C.Mikan	RSBV 98 (SPF)	São Paulo, São Paulo (Brazil)
Subtribe Tradescantiinae		
*Callisia repens* (Jacq.) L.	HUESC 15740	Ilhéus, Bahia (Brazil)
*Tradescantia cerinthoides* Kunth	Pellegrini 482 (RB)	Vacaria, Rio Grande do Sul (Brazil)
*Tradescantia fluminensis* Vell.	RSBV 96 (SPF)	São Paulo, São Paulo (Brazil)
*Tradescantia pallida* (Rose) D.R.Hunth.	HUESC 15737	Ilhéus, Bahia (Brazil).
*Tradescantia zebrina* Heynh. *ex* Bosse	RSBV 105 (SPF)	São Paulo, São Paulo (Brazil)
*Tripogandra diuretica* (Mart.) Handlos	UESC 20206	Ilhéus, Bahia (Brazil)
*Tripogandra warmingiana* (Seub.) Handlos	RSBV 104 (SPF)	Ilhéus, Bahia (Brazil)

All analyzed species were collected in University of São Paulo and during field trips in Brazil from 2014 to 2018. All species are being cultivated in the plant breeding facility of the Biosciences Institute, University of São Paulo. For all analysis, shoot apex less than 1mm high were colected from adult plants containing eight phytomeres from the stem apical meristem.

### Light microscopy analyses

Shoot apex of 14 selected species were fixed in FAA_50_ for 48 hours [15], dehydrated in an increasing ethylic series and included in methacrylate, according to Leica manufacturer's protocol, and in paraplast [16]. Transverse and longitudinal sections (4–10μm) were obtained on a rotating microtome. Samples included in methacrylate were stained with Ruthenium Red and counter-stained with Toluidine Blue [17], while the samples included in paraplast were stained with Astra Blue and Safranin [18]. Sections made by hand were obtained using a razor blade, and were stained with Alcian Blue and Fuchsin [19].

Anatomical analyses of the sections were performed under a light microscope and the images were obtained in the Image Scanning System (IM50) coupled with the Leica DMBL microscope (Leica Microsystems GmbH, Wetzlar, Germany) from Biosciences Institute, University of São Paulo. These images were imported to CorelDraw (software) for the graphic vectorization and manual segmentation. Each image was placed sequentially in a multilayer file. All images were vetorized, segmented and exported to tiff format file. The stack image created was imported to 3D reconstruction software to generate a 3D model.

The nomenclature adopted in the vascular bundles is shown in the Table 2, along with the terms used in the literature.

**Table 2:** Nomenclature for the vascular bundles. Major bundle (sympodial vascular bundle that constitutes the internal vascular system), Minor bundle (sympodial vascular bundle that constitutes the external vascular system), Cortical bundle (sympodial vascular bundle which cross, through the cortex, more than one internode), Central bundle (internal non-sympodial vascular bundle), Cauline bundle (external non-sympodial vascular bundle).

**Nomenclature adopted in the present study**	Guillaud (1878)	De Bary (1877)	Haberlandt (1914)	Scott & Priestley (1925)	Sharman (1942)	Esau (1943)	Fahn (1982)	Evert (2006)	Beck (2010)
**Major bundles**	“Faisceaux communs” (Common bundles)	Bundles of the leaf trace	Median bundles	Perimedullarbundles	Lateral or basifugal bundles	Larger bundles	Larger bundles	Major and large bundles	Major bundle
**Minor bundles**	“Faisceaux communs” (Common bundles)	Bundles of the leaf trace	Lateral bundles	Cortical bundles	Intermediate bundles	Smaller bundles of the leaf	Smaller bundles	Minor and intermediate bundles	Minor bundle
**Cortical bundles**	-	-	Cortical bundles	-	-	-	-	-	Cortical bundle
**Central bundles**	-	-	-	Medullary bundles	-	-	-	-	-
**Cauline bundles**	“Faisceaux caulinaires” (Cauline bundles)	Cauline bundles	Cauline bundles	Peripheral bundles	-	Smaller bundles of the stem	Common bundles	-	Cauline bundle

### Diaphanization

Shoot apex from the 14 species were fixed in 80% alcohol and placed in a heater at 45°C, with the alcohol replaced three times in a period of two weeks. To increase clarity of the samples, the material was immersed in 5% NaOH solution (replaced twice over a period of one week) and placed in a heater at 45°C. Subsequently it was rinsed in distilled water for 24 hours, clarified in chloral hydrate solution (1.6:1) for one week, and rinsed again in distilled water for 24 hours. The samples were then stained with Crystal Violet or Fuchsin and dehydrated in increasing series (50% to 95%) to remove excess dye [20].

### Analysis in X-ray microtomography

For this analysis, shoot apex (less than 1mm) of *Tradescantia zebrina* and *Dichorisandra thyrsiflora* were used. Samples were fixed in FAA_50_ for 48 hours, washed and stored in 70% ethanol [15]. These samples were scanned in a SkyScan 1176 X-ray Microtomograph (Bruker, Kotich, Belgium) of the Biosciences Institute, University of São Paulo. This analysis was performed under the following parameters: voltage (40kV), current (450uA), image rotation (-0.3630), rotation range (0.430°) and resolution (9μm). For the scans, samples were contrasted with Uranyl Acetate [21], Lead Citrate [22], Ruthenium Red (0.002% w/v H_2_O) [23], Phosphotunguistic Acid and Osmium Tetroxide (1% m/v in H_2_O) [24]. For the 3D reconstruction we used the programs CTVox and CTVol (https://www.bruker.com/service/support-upgrades/software-downloads/micro-ct.html), 3D Doctor (Able Software Corp., Lexington, USA) and ImageVis 3D [25], from which the image sequences were segmented automatically and manually.

### Analysis in confocal microscopy

Shoot apex of *Tradescantia zebrina* and *Commelina benghalensis* were stained with Aniline Blue and Berberine Hemisulfate [26] to detect callose deposits in sieve-tube elements and suberine or lignin in the secondary wall of tracheal elements. Images were obtained with LSM 880 confocal microscope (Carl Zeiss Microscopy, Jena, Germany). For Aniline Blue, the microscope was configured for excitation wavelength at 405 and detection wavelength at 415–502. For Berberine Hemisulfate the excitation wavelength was 488 and the detection wavelength 513–566. The samples were stained with 2 μg/ml solution of 4´,6-diamidino-2-phenylindole (DAPI) [27]. For DAPI, the microscope was set for excitation wavelength at 405 and detection wavelength at 398–502. For autofluorescence, the excitation wavelength was set at 488 and detection wavelength at 502–606.

Vectorization, whole-mount diaphanization, μCT and confocal microscopy were used to follow the course of vascular bundles and to indicate that vascular bundles have different trajectories.

## Results

### Classification of vascular bundles

**Fig 1:**
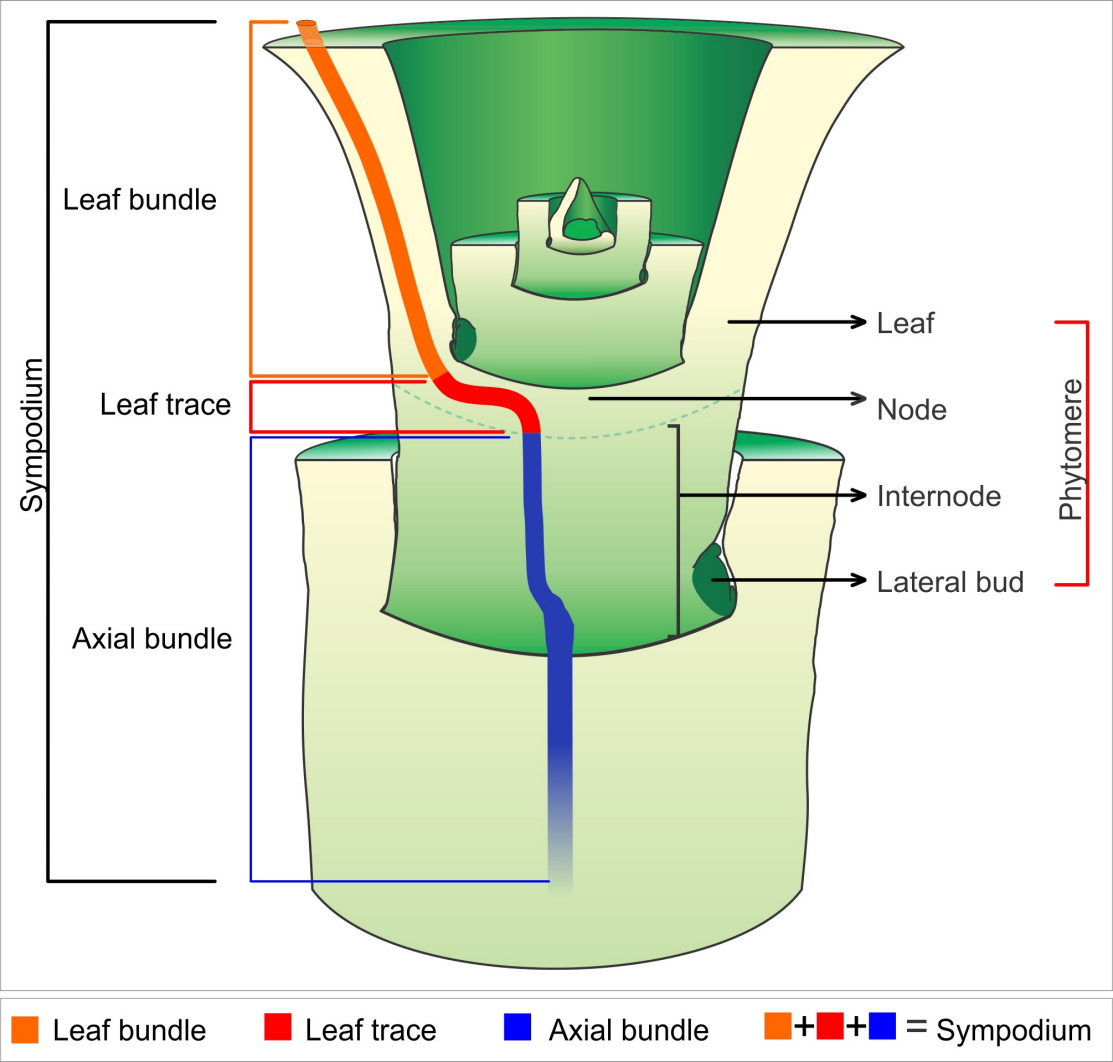
Illustration of a sympodium. Illustration indicating vascular region (i.e., leaf bundle, leaf trace and axial bundle), and also represents the division of the shoot apex into phytomeres. Each phytomere consists of leaf, node, internode and lateral bud.

**Fig 2:**
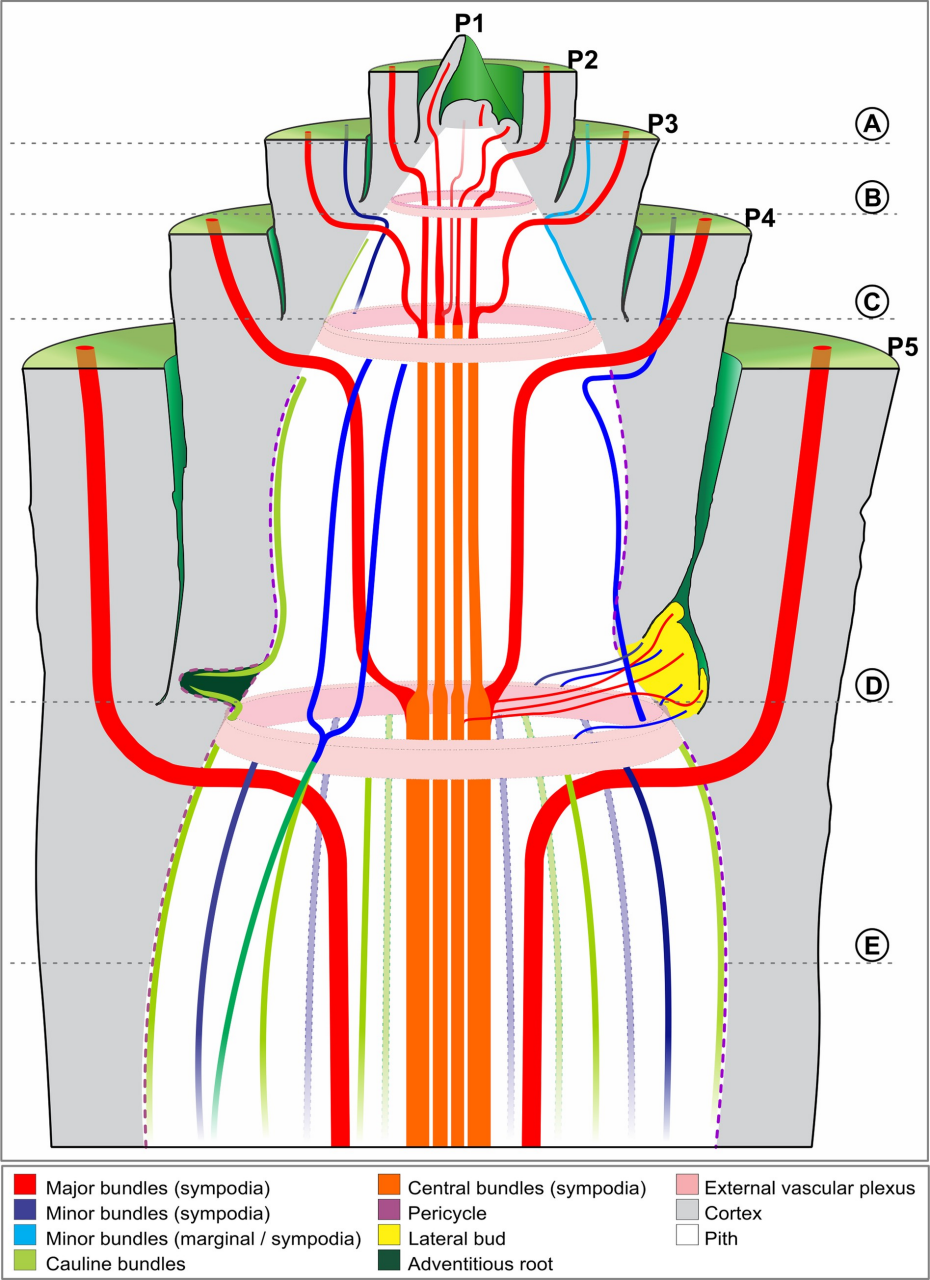
Illustration indicating the course of the bundles along the shoot apex and the connections of the nodal vascular plexus, leaf, bud and adventitious root. All major bundles (red) connect to the center of the vascular cylinder. The central bundles (orange) indicate the fusion of two or more bundles. The minor bundles have two similar trajectories. The first minor bundles (dark blue) run through the internode at a short distance from the pericycle (or continuous procambium with the pericycle), connecting to the vascular plexus of the node below, while the minor bundles of leaf margins (light blue) run through the internode adjacent to the pericycle (or continuous procambium with the pericycle). The other peripheral bundles (green) are the cauline bundles, which connect with each other or with minor bundles through the external nodal vascular plexus.

The vascular bundles can be divided into two general categories (Figs 1 and 2): 1) Cauline bundles–bundles that do not establish direct connections with the leaves (Fig 2); and 2) Sympodial bundles- continuous bundles between stem and leaf, which constitute the leaf traces (Fig 1). Leaf traces are the transition regions between the stem and leaf, where the leaf bundles connect to the axial bundles of the stem (Fig 1). The vascular bundles that constitute the sympodia can be subdivided into three categories: 1) Major bundles- first bundles to be originated, which occupy the medial region (between the peripheral bundles and central bundles) of the pith, along the internode; 2) Minor bundles- bundles that arise between the major bundles and occupy the periphery of the pith; and 3) Central bundles—of the pith, resulting from the fusion of major bundles in the internal nodal vascular plexus (IVP).

**Fig 3:**
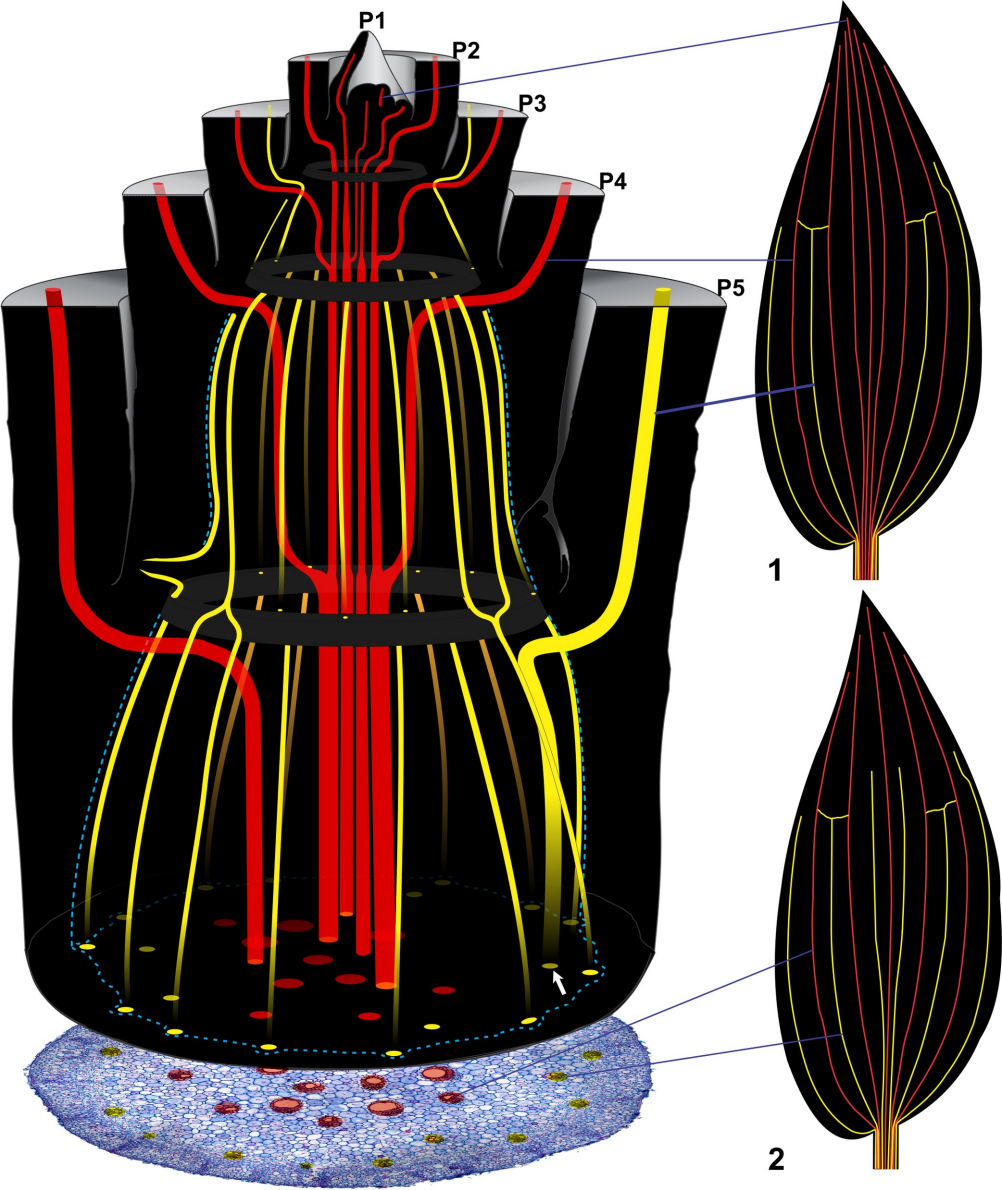
Illustration of a longitudinal section of the sympodium. Note division of vascular bundles in internal (red) and external systems (yellow). The internal system consists of major and central bundles. The external system is composed of minor and cauline bundles. Below the illustration, a transverse section of the 5^th^ internode of the shoot apex of *Commelina benghalensis* L. shows the distribution of systems in an internode. The illustrations of leaves 1 and 2 represent two patterns of leaf venation. In illustration 1, only four minor bundles occur. In illustration 2, six bundles developed between the major bundles, two of them on each side of the midrib. Note that in both the illustration and the transverse section of *C*. *benghalensis* the minor bundle (white arrow) is slightly away from the pericycle (blue dotted line).

Regarding the topology of the vascular system, two distinct patterns are recognized: internal and external vascular system (Fig 3).

1. Internal vascular system—constituted of sympodia (major bundle) and central bundles which connect in the internal region of the nodes, thus forming the IVP.
2. External vascular system—constituted of sympodia (minor bundle) and cauline bundle, which connect in the external region of the nodes to form the external nodal vascular plexus (EVP).

### Course of the vascular bundles

#### Major bundles

**Fig 4:**
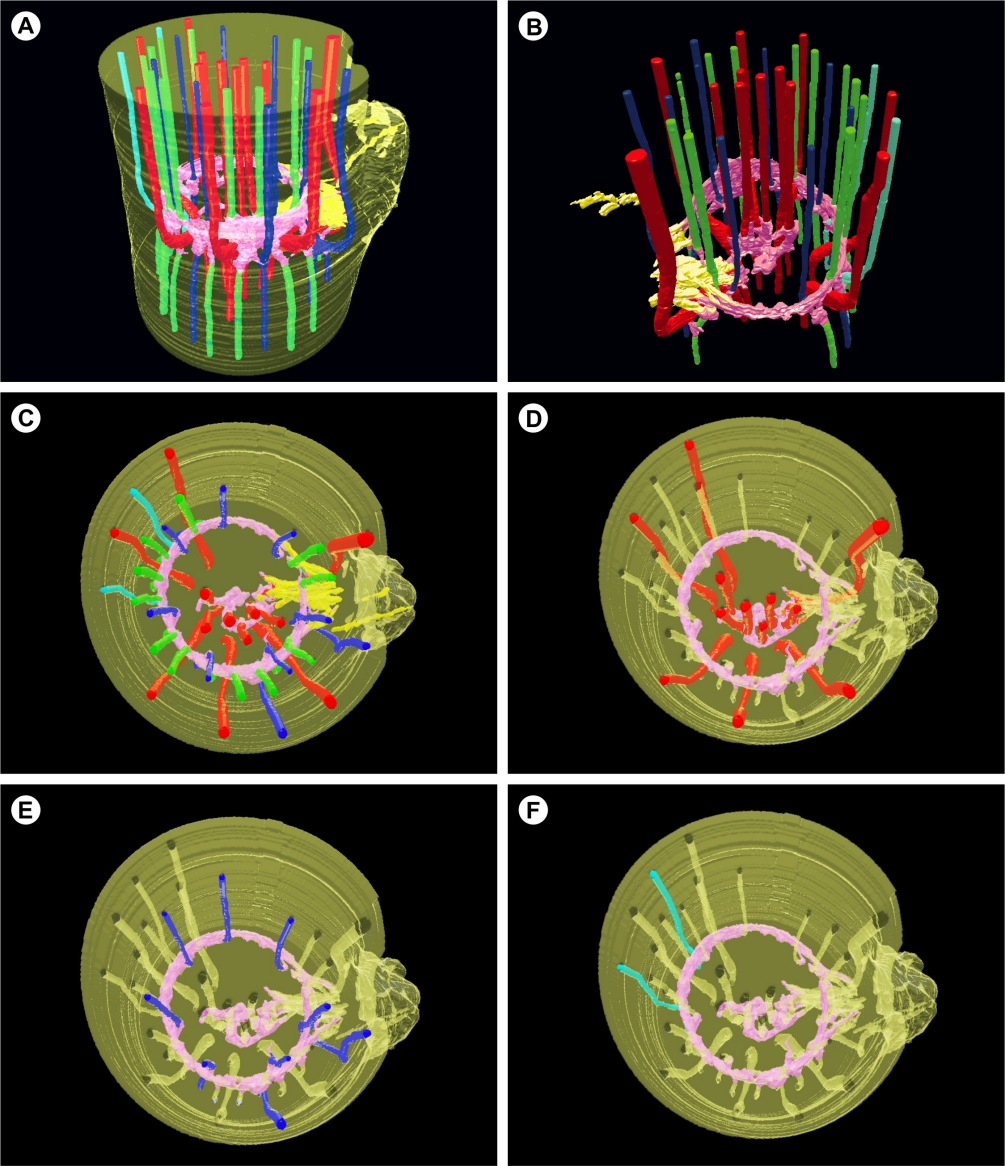
3D reconstruction through manual segmentation of bundles and automatic segmentation of the parenchyma. Image sequence of the nodal region of *Tradescantia zebrina* Heynth. ex. Bosse obtained in μCT. A) Rendered volume of all bundles, IVP and EVP. Major bundles and central bundles (red), minor bundles (blue), minor bundles of leaf margins (light blue), cauline bundles (green), lateral bud bundles (yellow) and internal and external nodal vascular plexus (light pink). B) Rendered volume only with vascular bundles and plexus. C-F) Transverse plane. C) Complete volume. D) Rendering only of the major and central bundles, plus plexus. Note that the EVP delimits the central cylinder, so the major bundles external to the plexus are in the leaf sheath. E) Rendering of the minor bundles plus plexus. F) Rendering of marginal minor bundles.

**Fig 5:**
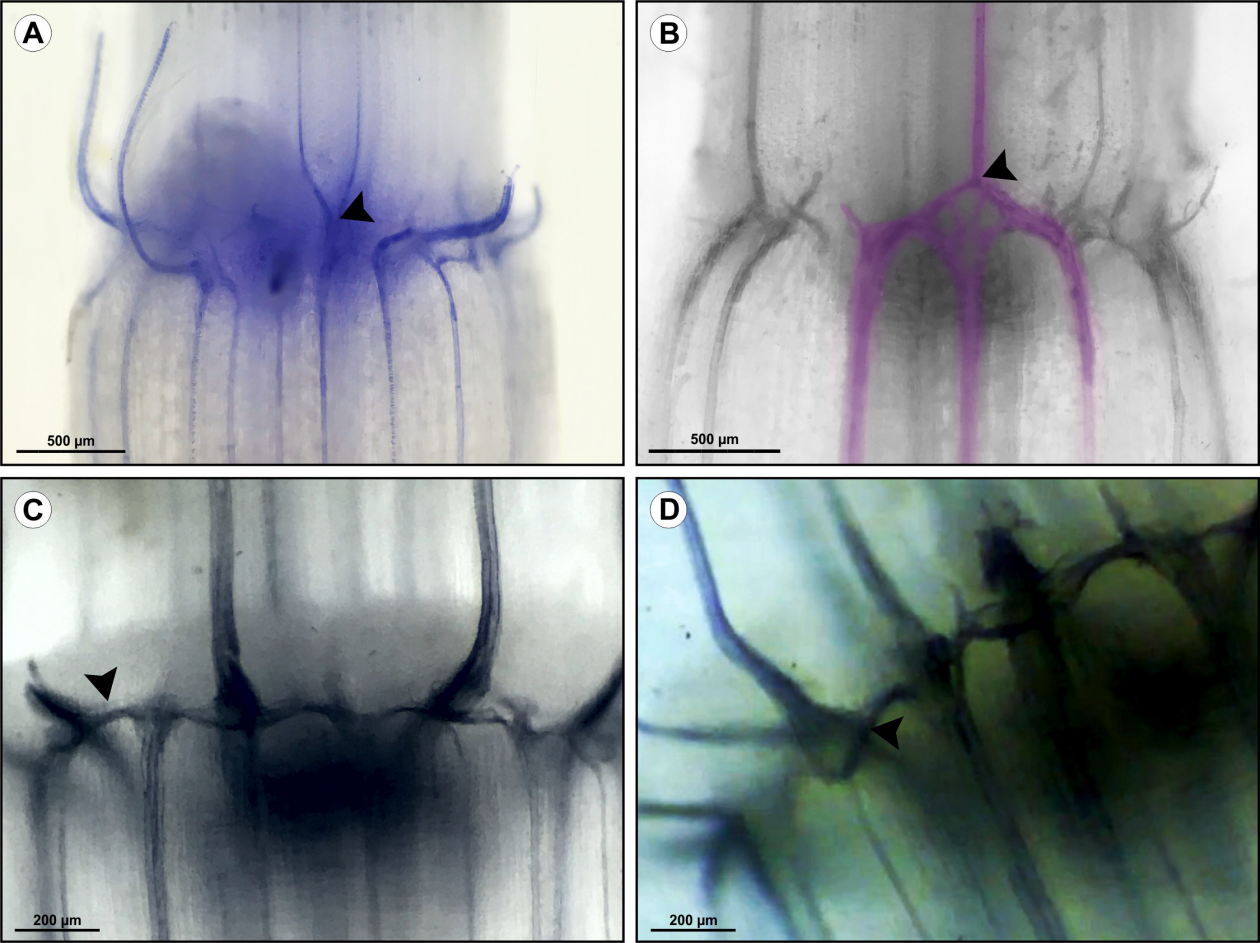
Whole-mount diaphanization. In *Tradescantia zebrina* Heynh. *ex* Bosse (A-B) and *Commelina benghalensis* (C-D). Note the bifurcation connecting two bundles from the internode above to a bundle from the internode below in figure A (arrowhead). The inverse situation can be observed in figure B, where a bundle of the internode above connects to two bundles of the internode below (arrowhead). Colored digitally. In figures C and D, it is possible to observe the transverse and oblique connections in the external nodal vascular plexus (arrowheads).

**Fig 6:**
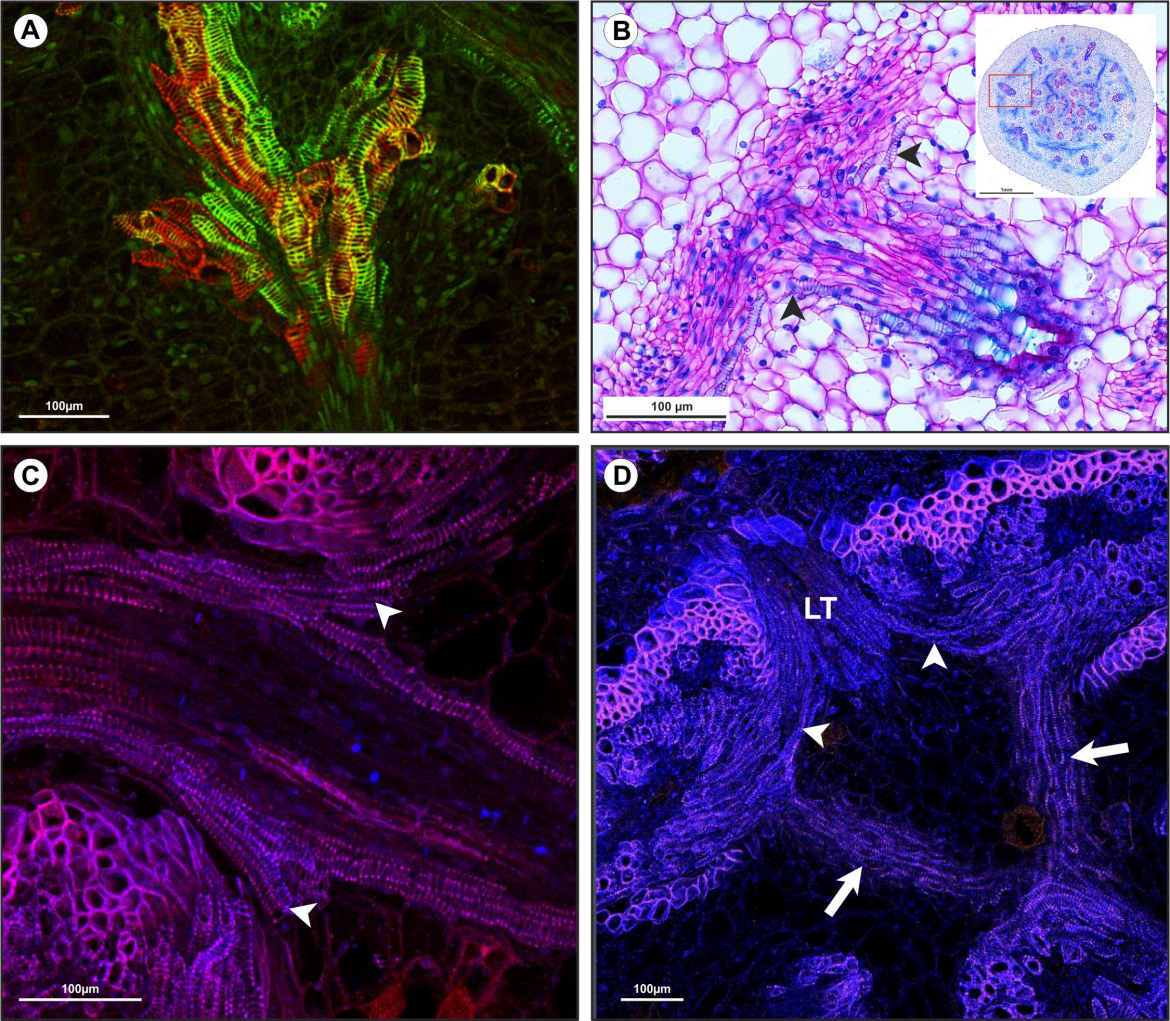
Analysis of vascular connections in the nodal vascular plexus region. A) Longitudinal section of *Tradescantia fluminensis* Vell. in the IVP region showing tracheal elements at different stages of differentiation. B) Transverse section of *Commelina benghalensis* L. showing connections between the leaf traces and peripheral bundles (arrowhead). C-D) Transverse section of *C*. *benghalensis* analyzed in a confocal microscope. C) Leaf trace between two vascular bundles. Contrast with aniline blue and berberine hemisulfate indicated callose plates in the leaf trace, but plates were not present in the oblique and transverse connections between vascular bundles. D) Connections between the IVP and the EVP (arrows), and connections between the leaf trace (LT) and peripheral bundles (arrowheads).

**Fig 7:**
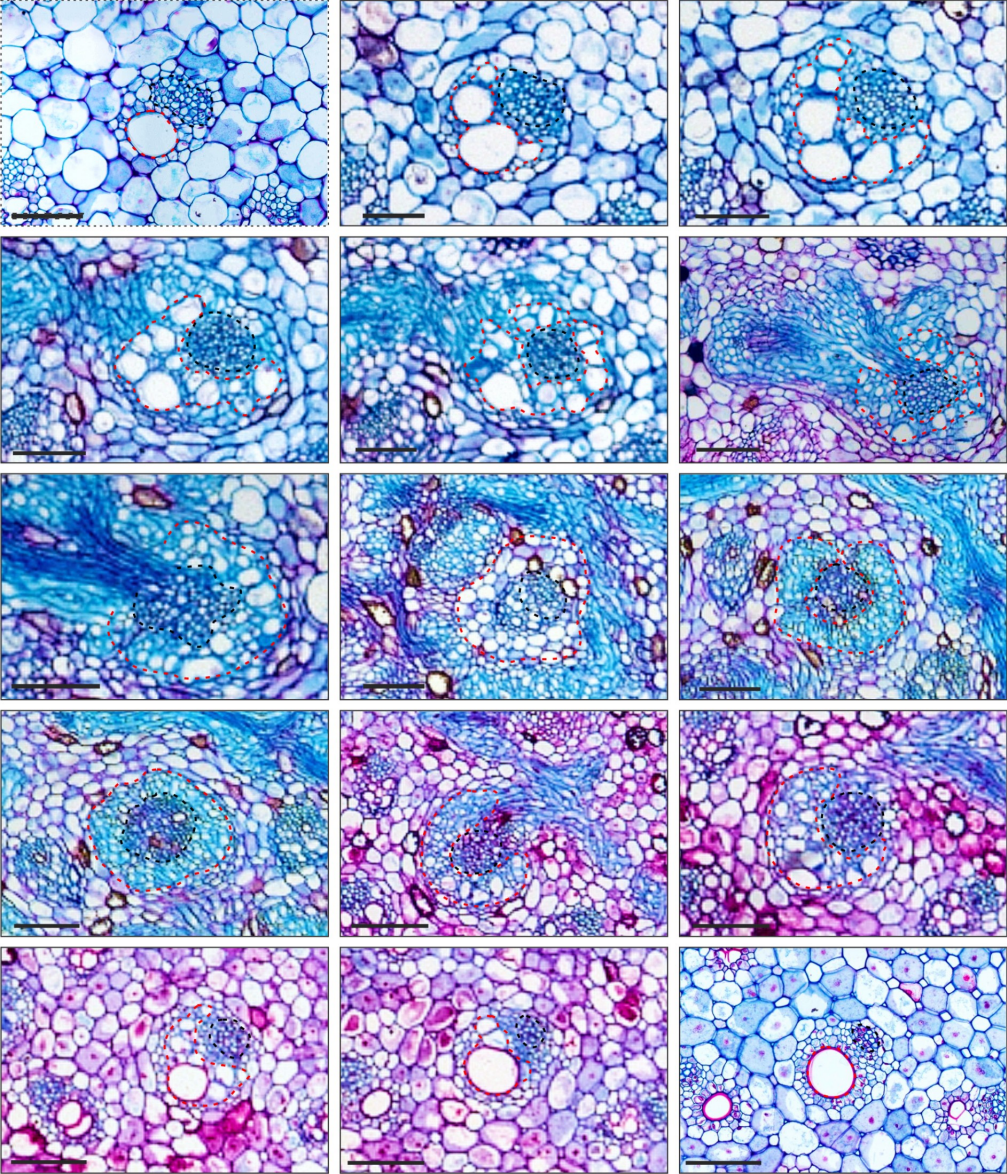
Anatomical variation of central vascular bundles. Image sequence in order from left to right and top to bottom, showing the anatomical variation of central bundles crossing the IVP. In the upper region the bundles are collateral, but along the IVP they change to an amphivasal arrangement, returning to collateral when the next internode starts below. Traqueary elements (red doted line) and phloem (black doted line). Scale bars 100μm.

Longitudinally these have a rectilinear trajectory along the leaf and its sheath, curving towards the center of the shoot in the region of the node to compose the leaf trace (Fig 2). After a short centrifugal trajectory in the region of the leaf trace, the major bundles reach the median region of the pith and then curve downward, starting a rectilinear descending trajectory to the base of the internode (Figs 2 and 4). Before reaching the base of the internode, the major bundles curve centripetally connecting to the IVP (Fig 2). At this point, two or more bundles can make connections between each other (Figs 4 and 5) or by means of tracheary element bridges differentiated from the intercalary meristem. The major bundles also connect to peripheral bundles of the EVP through tracheary elements (Fig 6). The number of major bundles that are connected to each other depends on the species. After the fusion of the major bundles in the IVP (Fig 2), the central bundles continue their downward rectilinear trajectory in the internode below. In the region of the nodes, most bundles lose their conventional collateral configuration, being able to acquire an amphivasal arrangement (Fig 7). The amphivasal bundles only occurs in the short space that comprises the internal vascular plexus, where the tracheary elements begin, gradually, to encircle the phloem (Fig 7).

#### Minor bundles

**Fig 8:**
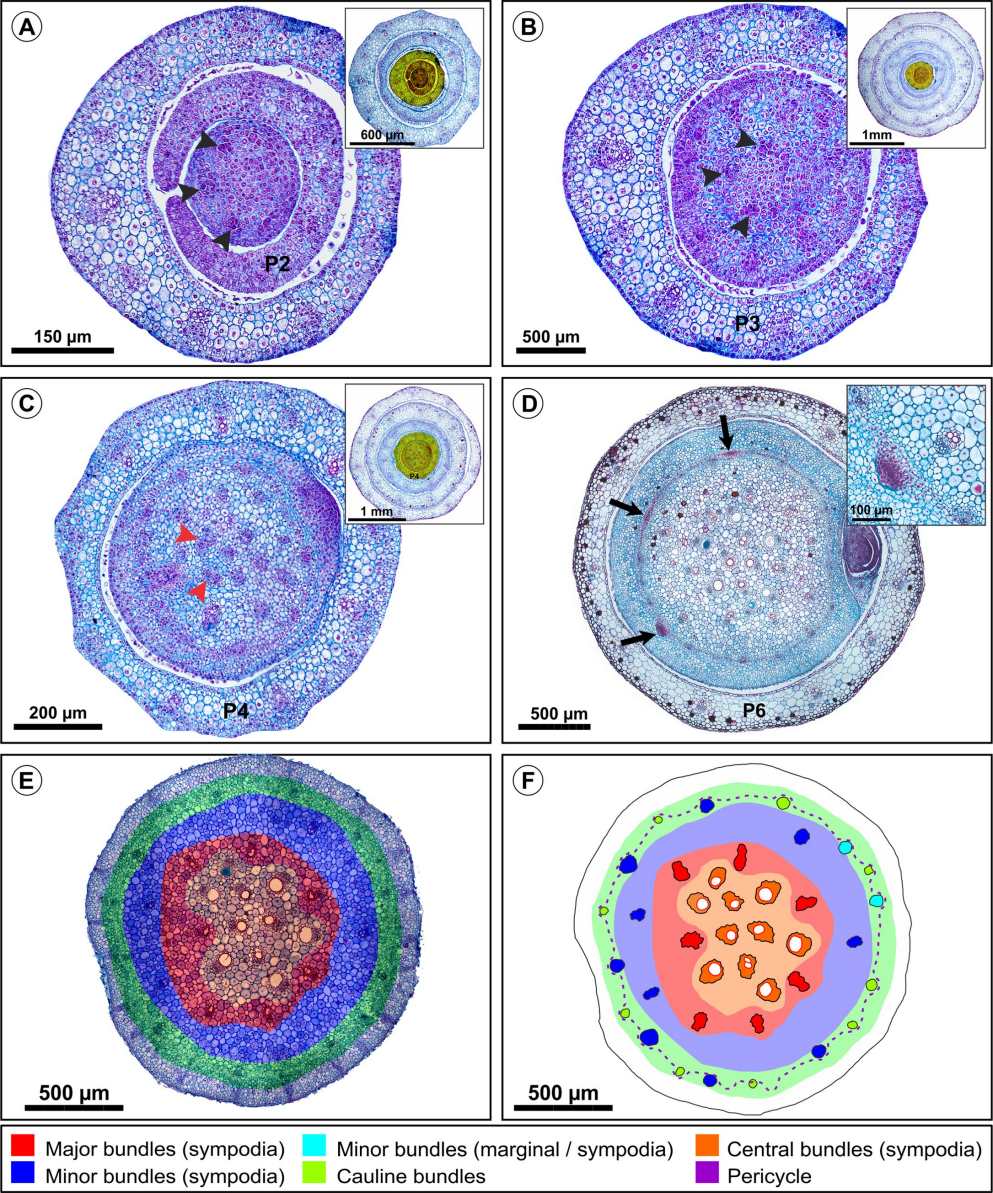
Transverse sections of the stem of *Commelina benghalensis* L. A) Nodal region of the second phytomere showing three unconnected procambial strands (black arrowheads). B) Nodal region of the third phytomere showing that there is still no fusion of the first three procambial strands (black arrowheads). C) Nodal region of the fourth phytomere indicating the first fusions of major bundles to the internal vascular plexus (red arrowheads). D) Nodal region of the fifth phytomere indicating primordia of adventitious roots. E) Internode region of the fifth phytomere showing digitally colored circles to indicate rings formed by each category of bundles. The most peripheral ring (green) consists of cauline bundles of the fifth phytomere and minor bundles from the fourth primordium. F) Illustration of figure (E) with bundles colored according to their respective category.

Their trajectory is similar to that of the major bundles, being axially rectilinear along the leaf and leaf sheath and curving into the stem in the node region to compose the leaf trace. However, before reaching the median region of the pith, they change direction to a centrifugal trajectory towards the periphery of the pith (Figs 2, 4 and 5B). This change in trajectory occurs at a very short distance from the nodal vascular plexus, giving the bundle, in longitudinal section, a pronounced curvature toward the periphery of the vascular cylinder. Thus, these bundles being closer the periphery of the vascular cylinder, but some of these maintain a small distance from the pericycle (Figs 4E and 8F and S4A Fig). Among the minor bundles, the two bundles adjacent to the leaf edges (Figs 2, 4F and 8F) have a different trajectory. These two bundles represent the last bundles originated, thus being opposed to the midrib. Unlike the other minor bundles, the ones on the border, as they converge to the stem, connect directly to EVP.

The differentiation of minor bundle is not uniform throughout the sympodium. The segment of minor bundle, which has a late differentiation, at the base of the internode, may remain structurally undifferentiated due to the activity of the intercalary meristem, or even differentiate later.

#### Central bundles

Located in the center of the pith, the central bundles establish connections between two IVP, and the course of these bundles is always rectilinear along the internodes.

#### Cauline bundles

These are located in the periphery of the pith, adjacent to the pericycle, with a rectilinear trajectory along the internode, although it does not form a leaf trace. They are alternated between the minor bundles and are slightly more external (Figs 2 and 8F).

### Distribution of vascular bundles in the internodes

**Fig 9:**
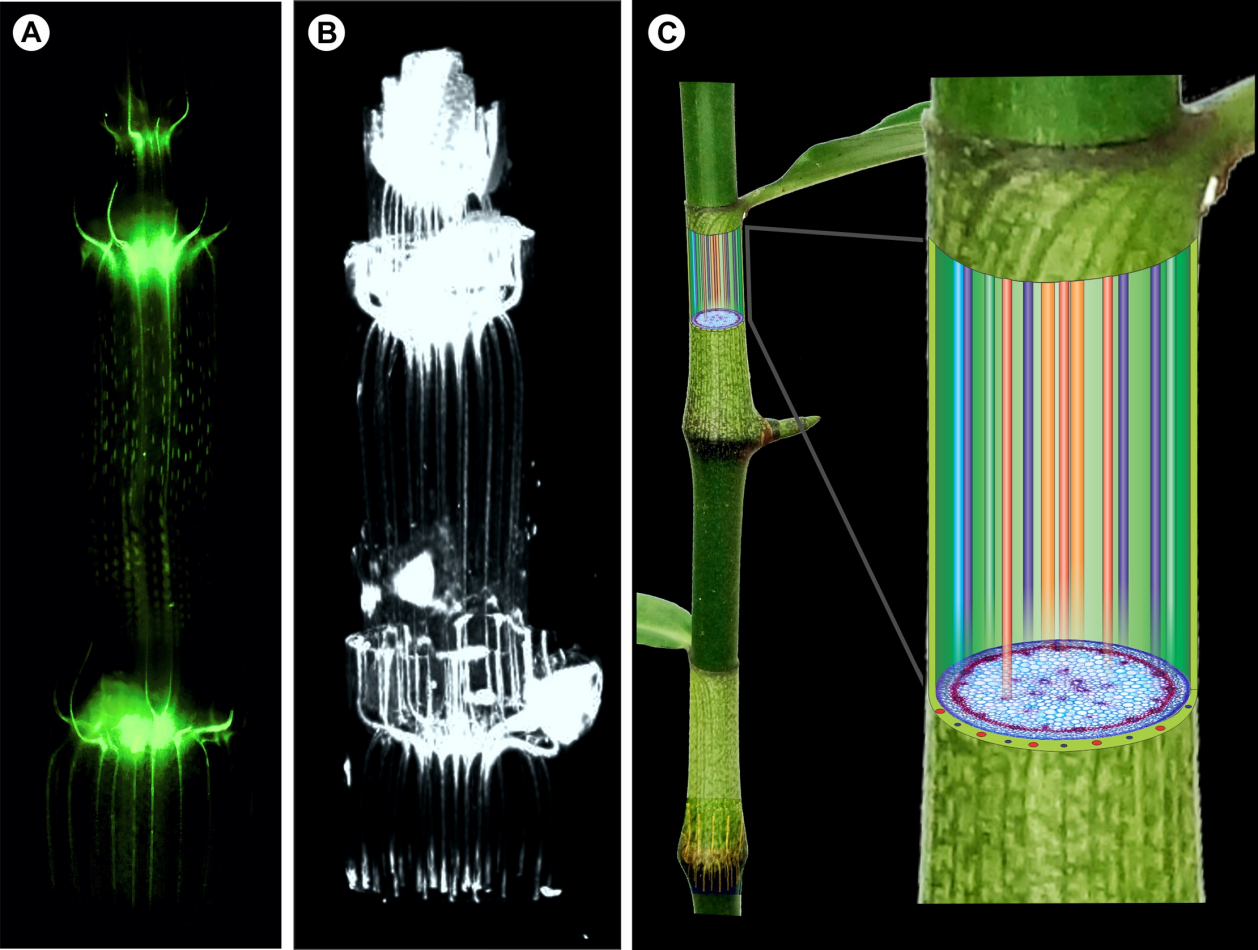
Overview of the course of vascular bundles along the main axis of the stem. A) Whole-mount diaphanization of the shoot apex of *Tradescantia zebrina* Heynh. *ex* Bosse. B) μCT analysis by contrast attenuation of the shoot apex of *T*. *zebrina*, contrasted with lead citrate. C) Illustration indicating the straight course of vascular bundles in the internode of *Dichorisandra thyrsiflora* J.C. Mikan.

The distribution of vascular bundles in the internodes presents little variation between the analyzed species. This variation is related to the number of bundles and the nodal vascular plexus pattern (Figs 4, 8E–8F and 9).

In transverse section, vascular bundles are concentrically organized in the pith, that is, in circumscribed bundle rings (Fig 8E and 8F). Each ring is related to a category of vascular bundle as to its origin and course. Except in the first three internodes of the shoot apex, any internode of the analyzed species presents at least one representative of each type of bundle.

The central ring (Fig 8E and 8F) consists of axial bundles that start from an IVP and run through the entire length of the internode until they connect to the IVP of the next node (Fig 2, S7A and S8A Figs). The median ring is formed by major bundles around the central bundles (Fig 8E and 8F). At least one major bundle passes through the IVP without any connection (S4E, S6B and S7B), generally with the first bundle corresponding to the midrib of the leaf.

### Vascular connections in the nodal vascular plexus region

The major, minor and cauline bundles run freely through the entire length of the internodes, with no connection between bundles during this path (Figs 2 and 9). On the other hand, in the nodal region, an anastomosed network of tracheary elements continue to differentiate from the intercalary meristem (including the pericycle). This network connects major bundles in the center of the pith (Figs 4 and 6A and S8A Fig) and minor and cauline bundles on the periphery of the pith. These connections are oblique or transverse, both in the IVP and the EVP (Fig 5B and 5D).

The EVP connects the minor vascular bundles to the cauline bundles, or two cauline bundles. On the other hand, the major and central bundles connect on the IVP region forming three different patterns. In the first case, once fused, they are concentrated in a central circle (Pattern 1, S6 Fig). Another pattern, which is similar to the first, presents the IVP divided into two vascular columns (Pattern 2, S7 Fig). In addition to the connections of the first and second patterns, connections between the EVP and IVP (Pattern 3, S8 Fig) may also occur, depending on the species analyzed. In the first and second patterns (S6 and S7 Figs), there is no connection between the IVP and the EVP and at least one major bundle passes freely through the IVP (S4E, S6B and S7B Figs). Connections between the vascular system of the stem/leaf (i.e., leaf traces), the lateral buds and the adventitious roots are established by the nodal vascular plexus.

#### Leaf trace

The major and minor vascular bundles establish distinct connections in the region of the leaf trace. Until the third phytomere it is possible to observe minor bundles passing freely through the EVP, because at this stage of development the tracheary elements are not yet differentiated (Fig 8A and 8B). The minor bundles passing through the EVP establish oblique or lateral connections through tracheary elements (Fig 5). These connections form bifurcations, connecting one bundle from the internode below to two bundles from the internode above (cauline bundles) (Fig 5A), or two bundles from the internode below to one bundle from internode above (minor bundle) (Fig 5B).

When the major vascular bundles and central bundles approach the node below, there is an interruption of the vessel, and its terminal region connects to tracheary elements of the IVP (S5A and S5B Fig). Likewise, when initiating the next internode, the same connections occur with vessel elements of the central bundles that run through the internode below (S5B Fig). Thus, these vascular connections of the IVP are directly related to the organization of the central bundles, which present a metaxylem with a larger diameter and absence of protoxylem (S5C Fig). The central region of the pith is generally composed of six central bundles, but may vary between 2 and 11 bundles, depending on the species (S5D and S5E Fig).

In the nodal region it is possible to verify that leaf traces form leaf gaps, both for the major and minor bundles (S5F Fig).

#### Lateral bud / lateral branch

In the same way as the vascular development of the shoot apex, the first bundles to develop in lateral buds are the major bundles. The internal vascular system of the buds connects to the stem’s IVP, a connection that can be observed in the initial stages of these buds (S4D and S9A and S9B Figs). In later stages of bud development (approximately at the 4^th^ lateral bud from the apical meristem), when the minor bundles have appeared, it is possible to observe that the external vascular system of the buds also connects to the external vascular system of the main shoot axis through the EVP (S9C Fig).

#### Adventitious root

The adventitious roots ony connect to vascular bundles of the EVP (S9D Fig).

## Discussion

### Nomenclature of vascular bundles

Since the 19^th^ century, with the beginng of vascular architecture studies in plants, there is great inconsistency when naming and recognizing the types of bundles. This has generated conflicting interpretations. In general, vascular bundles can be continuous between the leaf and the stem, or discontinuous and existing only in the stem, as observed in species of Commelinaceae. While in the Commelinaceae the leaf trace perpendicularly crosses the cortex, in other monocotyledons the leaf trace may cross, through the cortex, more than one internode before connecting to the vascular system of the stem. A particular feature of some monocotyledons is the presence of a discontinuous cortical bundle, which may accompany a leaf trace [4]. The cortical bundles are sometimes leaf traces, which run through the internode for some distance outside the vascular cylinder before curving into it [6]. In the gymnosperm *Cycas revoluta* Thunb. (Cycadaceae), the presence of a bundle passing through the cortex was observed, but the term cortical bundle was not used by the author [13].

The region of the sympodium, which connects the stem bundle to the leaf bundle, is called a leaf trace [28]. When it crosses more than one internode, through the cortex, before converging to the stem, it is called a cortical bundle [29]. Based on our findings in Commelinaceae, it is clear that cortical bundles [4, 6] are actually leaf traces, which cross, through the cortex, more than one internode before connecting to the vascular system of the stem.

Most authors do not mention cortical bundles (Table 2), as they were not present in the plants they analyzed. Cauline bundles were observed by some authors, but were not named. In *Zea mays* (Poaceae), large amounts of peripheral bundles were visualized, some of which were continuous with intermediate bundles [30]. Bundles that do not connect with the leaf traces also occur at the periphery of the vascular cylinder. However, these bundles have not yet been named [30]. Similarly, it was shown in a longitudinal section of *Z*. *mays*, vascular bundles (considered by the author to as leaf traces) crossing the entire longitudinal lenght in the peripheral position [5]. In Commelinaceae, the peripheral bundles whose course is entirely peripheral, also do not connect to the leaf traces and are denominated as cauline bundles, in the present study. Cauline bundles might present only metaxylem and metaphloem [7, 31, 32] or present metaxylem, metaphloem, protoxylem and protophloem, as shown in this study. In some cases, this divergence in nomenclature can take place in the same specimen. For instance, in *Z*. *may*s (Table 2), where the course of the major bundles is vertical and does not approach the center of the pith [30], or where the major and minor bundles are horizontal in the region of the nodes and approach the center of the pith [5].

The Table 2 presents the main terms used in the classification of vascular bundles based on their course and distribution. In the species analyzed in this study, it was verified that bundles originated in the first three primordia are always continuous between stem and leaf (sympodial), denominated major bundles following the nomenclature of some authors [4, 5, 28, 33]. As for the minor and cauline bundles described in the present study in Commelinaceae, we follow the concept already adopted by several authors [1, 2, 4, 6, 7, 34] in different monocotyledon families, including Commelinaceae. The fourth bundle category found in the analyzed species was called central bundle, which results from the fusion of major bundles in the IVP. This bundle is described here for the first time, and to our knowledge, have never been referenced it in the available literature.

### Distribution of vascular bundles in the internodes

As previously shown, vascular bundles are classified according to their course along the shoot axis in major bundles, minor bundles, central bundles, and cauline bundles. However, the nomenclature of the bundles is established based on their relative position in a transverse section of the internode (i.e., peripheral, central, medullary, perimedullary, cortical, etc.) and bundle size (i.e., major, minor, intermediate, etc.). The vascular bundle nomenclature utilized for *Tradescantia fluminensis* was based on their position [12]. According to these authors, the bundles positioned in the periphery were called peripheral, the pith bundles were called medullary, and the bundles located in the perimedullar region (between the peripheral and medullary) were called perimedullary.

Despite anatomical and morphological differences between the different monocotyledon families, the distribution of bundles and their nomenclature can be quite similar. In transverse sections of *Zea mays* (Poaceae), minor bundles were reported at the periphery of the pith and major bundles were concentrated at the pith’s most central position, while other major bundles occupied an intermediate position between the central and peripheral bundles [5]. This configuration is the same as that found in the herein studied species of Commelinaceae, where minor and cauline bundles are on the periphery of the pith, major bundles on the pith’s median region (perimedullar) and the central bundles in the innermost region of the pith. Some plants, like *Scleria bracteate* Cav. (Cyperaceae), can present a different configuration, where the cauline bundles are placed at the center of pith [31].

In transverse sections of the internode of Commelinaceae, bundles are concentrically organized, although they are not perfectly aligned in their respective layers. The order of bundles is established according to their course and specific distribution, where each type of bundle (i.e., major, minor, central, and cauline), in transverse section, occupies a circumscribed layer. In most monocotyledons, the bundles are considered to be "scattered" in the pith. However, in species with long internodes and whose procambium is similar to that of eudicots, the bundles may be arranged in concentrical rings [13].

In this study, it was possible to track with a certain degree of precision the course of vascular bundles of the Commelinaceae, and to compare it with the data available in the literature. According to previously published studies, in general, two or three groups of vascular bundles are described based on their course and position in a transverse section of the internode. Through three-dimensional analysis, it was possible to verify a new category of bundles (i.e., central bundles), as well as the result of fusion between bundles in the IVP.

### Classification of bundles based on their system or connection with the nodal vascular plexus

Studies of the vascular system in many monocotyledons have revealed that these plants have two distinct systems of vascular bundles, one internal and one external [3, 8, 35]. The inner system consists of leaf traces connected to the axial bundles of the stem, while the external system consists of bundles that “end blindly” in the cortex [3]. In the species herein studied, two systems were also verified: 1) the internal, constituted of major bundles, central bundles and IVP; and 2) the external system, constituted of minor bundles, cauline bundles and EVP.

The vascular system might also be classified according to the connections between bundles and their continuity along the shoot axis. When the plant has a vascular system composed of bundles that form leaf traces and independent bundles that do not connect with the leaves (i.e., cauline bundles), the vascular system of this species is considered open [3, 28]. Based on this statement, the vascular system of Commelinaceae could be considered open due to the presence of the cauline bundles. Nonetheless, the cauline bundles of the analyzed species are connected to other cauline bundles and to minor bundles by means of tracheary elements in the region of the EVP. In some species, like *Commelina benghalensis*, leaf traces also discreetly connect to the EVP by mean of tracheary elements. In summary, from the combined analyses performed in this study, we suggest that there is no open vascular system since all bundles have some degree of connection along the shoot axis. In Arecaceae, similar conections are made by mean of bridges, that are short vascular branches which connect to the neighboring axial bundle [8]. In Velloziaceae, it was observed that the metaxylem elements diverge from leaf trace and each divergent metaxylem was connected to each flanked cauline bundles [7].

### Course of vascular bundles

The course and distribution of vascular bundles establishes the vascular architecture of plants, reflecting the intimate relationship between leaves and stem. This course is similar in some monocotyledon families (e.g., Acoraceae, Araceae, Poaceae, Costaceae, Dioscoreaceae, and Smilacaceae). In general, there are only two basic types of bundles: 1) those that are continuous between stem and leaf; and 2) the cauline bundles that are not continuous with the leaves [6].

Generally, in the nodal region, more than one bundle in the shoot diverges from the vascular cylinder in the stem towards the leaf, with major bundles being the ones closest to the center of the pith, while minor and cauline bundles longitudinally run along the pith’s periphery [2, 3, 6]. The sinuosity of these bundles in longitudinal section is generally described either as: 1) convergent, in relation to the connection of the leaf bundle to the leaf trace at the base of the leaf (from the leaf to the stem); or 2) divergent, in relation to the connection of the axial bundle to the leaf trace (from the stem to the leaf) [3, 4, 6]. In the studied Commelinaceae, the course of the bundles also present centripetal and centrifugal trajectories. Which means that the parameter used to define these trajectories is also related to the convergent or divergent condition of the bundles. In most studies, the trajectory is described as centrifugal, or towards the center of the stem, even when it is assumed that the bundles diverge from the vascular cylinder towards the leaves [2, 5]. In the species analyzed in the present study, the peripheral and minor bundles present a sharp curvature towards the center of the stem. This curvature is a consequence of the expansion of the vascular cylinder during the transition between two internodes.

The increase in height of the adult shoot in monocotyledons is a consequence of the intercalary meristem activity at the base of the internode [3, 4, 28]. Therefore, due to the early differentiation of the major bundles, the vascular bundle region at the base of the internode remains structurally undifferentiated. As the intercalary meristem remains active after the early structural differentiation of the major bundles, it is possible to see the basal region of these bundles still structurally undifferentiated. In the vascular system of *Tradescantia albiflora* Kunth (= *T*. *fluminensis*) the bundles are interrupted due to the intercalary meristem activity [36]. The basal region of this bundle, which has a late differentiation, is usually invisible when using conventional techniques, being misinterpreted as bundles ending in a “blind spot” [3] or “interrupted” [36]. Also, it was shown at the shoot apex of *Zea mays* [37], the lateral bundles of the young leaves and the stem are discontinuous in the early stage of their procambial differentiation, but become connected to each other latter on. Thus, procambial differentiation seems to be delayed at the node, resulting in discontinuous differentiation [37]. Simultaneously, the xylem can also be differentiated acropetally and unidirectionally in the intercalary meristem of the base of the internodes [38].

In the analyzed species, this region represents the base of the internode and consequently the same region of the intercalary meristem. The analysis of these bundles at the base of the internodes revealed that, due to the structurally undifferentiated state of the tracheary elements, conventional techniques in plant anatomy do not allow the visualization of these bundles in this region, which gives the impression that these bundles “disappear” at a certain point, at the periphery of the pith. However, when comparing the analyzes of μCT and whole-mount diaphanization with the serial transverse sections it was possible to verify that these bundles do not end blindly, but connect to the EVP.

It was reported in *Tradescantia fluminensis* that internode elongation causes rupture of tracheary elements of the medullary (named herein as central bundles) and perimedullary bundles (major bundles) [12]. In *Zea mays* (Poaceae), during an experiment designed to visualize the flow of eosin through conductive tissues, it was demonstrated that there was no rupture of tracheary elements in the intercalary meristem region, similar to that observed in the Commelinaceae species studied herein [5].

The cauline bundles and minor bundles are peripheral and have late differentiation, starting approximately at the third phytomere, after the major bundles are already differentiated. The late differentiation of some vascular bundles was documented in *Prionium serratum* (Thurniaceae) [39]. In this study, it was suggested that this delay in the differentiation process was the reason why these bundles failed to connect to the axial shoot vascular system, since they were outside the meristematic limit. This process was also observed in species of Commelinaceae, where major bundles were the only ones present in the first two phytomeres, thus constituting the internal vascular system. Nonetheless, this does not appear to be the reason why the minor and cauline bundles do not connect to the internal system, since the lateral buds establish vascular connections with both internal and external systems at the same time as these bundles begin to differentiate. Similarly, adventitious roots also establish connections between newly formed tissues and those already differentiated.

### Conections and patterns of the nodal vascular plexus / Types of bundle and anatomical variations

The distribution of vascular bundles is directly related to the presence of a vascular plexus, which characterizes the nodal region, where the connection of vascular tissues occurs [30, 40, 41]. Despite knowing of the existence of two distinct regions of the vascular plexus (IVP and EVP), it is still unclear, in the literature, of the existence of connections between these two systems. In *Tradescantia albiflora* (= *T*. *fluminensis*), connections were not found between the EVP and IVP [6]. However, the presence of vascular connections between the two plexus was observed in *Tradescantia fluminensis*, in a different study [12]. When fully differentiated, the peripheral bundles connect to the nodal ring, and an anastomosis of bundles arises connecting the external nodal ring to the central nodal plate [12]. Among the species analyzed in the present study, three nodal vascular plexus patterns have been identified. In Pattern 1 and Pattern 2 there was no connection between the IVP and the EVP, as in *Tradescantia*, *Tripogandra* and *Callisia*, while in Pattern 3, there are connections between the two plexus, as in *Commelina benghalensis*. Regardless of the differences between the three nodal vascular plexus patterns, the connections in the IVP and EVP link, to some degree, all major, minor and cauline vascular bundles.

The major bundles are collateral, but when they merge with the IVP, they form the central bundles of the internode below. In this region, the bundles present alterations in their organization and constitution. The tracheary elements of major bundles are connected to the IVP, through tracheids, which are organized around the phloem, acquiring an amphivasal arrangement. Just below the IVP the bundles return to their collateral arrangement, but only with metaxylem element. The change from collateral to amphivasal or amphicrival arragment was described in the nodal region of Acoraceae [13], Araceae [13], Cyperaceae [29, 42] and Poaceae [42]. In these families, it is described that the congestion of leaf traces in the nodal region is the cause of the alteration in vascular bundle arrangement. The amphivasal condition is directly related to fusion of the vascular bundles in the nodal region, where the IVP occurs. This condition is more expressive as the nodes are closer to each other (e.g., in rhizomes) [42]. In Commelinaceae, the amphivasal arrangement is restricted to the IVP in the nodal region and might be considered a transitory condition.

The correlation between the plexus region and amphivasal arrangement is also supported by the vascular bundles of Arecaceae and Zingiberales, in which neither a vascular plexus or amphivasal bundles can be observed [42]. It is worth mentioning that in Arecaceae there is no fusion of bundles in the nodal region, being added at each internode. The same process can be observed in Apiaceae and Araliaceae (Apiales), which lack a nodal vascular plexus, but present an amphivasal arrangement due to the numerous leaf traces in the nodal region [42].

The reduced number of vascular bundles in Commelinaceae does not prevent the establishment of an amphivasal arrangement, since congestion of leaf traces would be a condition for the emergence of this type of arrangement [42]. The change in arrangement of a collateral bundle to amphivasal may arise from the congestion of vascular bundles in the nodal region in some groups, such as *Acorus* spp. (Acoraceae) and *Dracena* spp. (Asparagaceae) [13]. The amphivasal bundles may be a consequence of the arrangement of the xylem (V-shaped), thus arising without the fusion of separate bundles [13]. In some species of *Tradescantia* (e.g., *T*. *cerinthoides*, *T*. *fluminensis*, and *T*. *zebrina*), despite the xylem in the major (colateral) bundles being V-shaped, they still merge to the nodal vascular plexus to acquire an amphivasal arrangement.

## Conclusion

Three-dimensional analysis techniques were fundamental to understanding the vascular architecture, especially regarding nodal vascular plexus connections. Our results show that the nodal vascular plexus connections are highly specialized in the Commelinaceae, in which bundles that end in a "blind spot" (considered interrupted) are a result of its late differentiation from the intercalary meristem. Tree different patterns of nodal vascular plexus were, so far, found in the Commelinaceae. Nonetheless, only additional sampling of the missing lineages its systematic relevance in the family might be properly accessed. The first description of central bundles in the Commelinaceae might suggests their existence in closely related groups, such as the remaining four families of Commelinales (i.e., Haemodoraceae, Hanguanaceae, Philydraceae, and Pontederiaceae), and even in other distantly related groups of monocotyledons. Further studies also sampling the Haemodoraceae, Hanguanaceae, Philydraceae, and Pontederiaceae are needed in order to properly test the putative systematic relevance of vascular architecture for the order and each of its families.

## Supporting information

S1 Fig Morphology and habit. Habit of *Aneilema beniniense* (P.Beauv.) Kunth (A), *Callisia repens* (Jacq.) L. (B), *Commelina benghalensis* L. (C), *Commelina rufipes* var. *glabrata* (D.R.Hunt) Faden & D.R.Hunt (D), *Dichorisandra radicalis* (E), and *Dichorisandra thyrsiflora* J.C.Mikan (F). Arrow in figure A and F indicate the pseudopetiole.
(TIF)

S2 Fig Morphology and habit. Habit of *Floscopa glabrata* (Kunth) Hassk. (A), *Floscopa* aff. *glabrata* (B), *Tradescantia cerinthoides* Kunth (C) and *Tradescantia fluminensis* Vell. (D), *Tradescantia pallida* (Rose) D.R.Hunt (E), and *Tradescantia zebrina* Heynh. *ex* Bosse (F). Arrow in figure B indicating pseudopetiole.
(TIF)

S3 Fig Morphology and habit. Habit of *Tripogandra diuretica* (Mart.) Handlos (A) and *Tripogandra warmingiana* (Seub.) Handlos (B).
(TIF)

S4 Fig Details of the vascular bundles along the stem axis. A) Transverse section of *Commelina benghalensis* showing the position of the minor vascular bundle, slightly away from the pericycle. B) Whole-mount diaphanization showing the late differentiation of a vascular bundle at the base of the internode, intercalary meristem region. C) Longitudinal section of *Tripogandra* sp. showing the continuity of a central bundle between two IVPs (black arrows). D) μCT analysis by contrast attenuation of the shoot apex of *Tradescantia zebrina*, contrasted with uranyl acetate. White arrow indicates connection of the internal vascular system of the lateral bud (LB) to the IVP. Red arrow indicates a leaf trace. E) 3D reconstruction by means of manual segmentation of the vascular system showing a major bundle (red) passing through the IVP without connections.
(TIF)

S5 Fig Connections and variations in vascular bundle structure in the nodal vascular plexus region. A) Whole-mount diaphanization of the nodal region of *Commelina benghalensis* indicating a terminal vessel element of the central bundle connecting to the IVP through tracheids (arrow). B) Longitudinal section of *Tripogandra warmingiana* in the IVP region showing the connection of major bundles to the IVP (arrows). C) Transverse section of the internode of *C*. *benghalensis* showing a central bundle only with metaxylem. D) Transverse section of *T*. *warmingiana* showing only two central bundles (arrowheads). E) Transverse section of the internode of *C*. *benghalensis* with ten central bundles (arrowheads). F) Whole-mount diaphanization of the nodal region of *C*. *benghalensis* indicating leaf gaps (arrowheads).
(TIF)

S6 Fig Pattern 1 of the nodal vascular plexus. A) Longitudinal section of *Dichorisandra radicalis* showing the median region of the IVP. B) Diaphanization of the nodal region of *Tradescantia zebrina* showing the concentric configuration of bundles in the IVP without connection to the EVP. Arrow indicates the major bundle that crosses the node without connection to the IVP. C) Transverse section of *Callisia repens* with concentric configuration of bundles in the IVP without connection to the EVP.
(TIF)

S7 Fig Pattern 2 of the nodal vascular plexus. A) Longitudinal section of *Tripogandra diuretica* showing the median region of the IVP. B) Diaphanization of the nodal region of *T*. *diuretica* showing the concentric configuration of bundles in the IVP forming two groups of bundles. C) Transverse section of *T*. *diuretica* in the nodal region showing the major bundle that crosses the node without connection to the IVP (arrow).
(TIF)

S8 Fig Pattern 3 of the nodal vascular plexus. A) Longitudinal section of *Commelina benghalensis* showing the median region of the IVP. During the assembly process of the shoot apex samples in stubs for scanning electron microscopy, the parenchyma cells absorbed moisture faster than vascular tissue, causing a depression that marked the vascular system throughout the sample. Vascular bundles and plexus of the left meridian were digitally colored. Arrow indicates EVP. B) Diaphanization of the nodal region of *C*. *benghalensis* showing the concentric configuration of bundles in the IVP forming two groups of bundles. Arrow indicates the major bundle that crosses the node without connection to the IVP. C) Transverse section of *C*. *benghalensis* in the nodal region, showing connections between the IVP and the EVP (arrowheads).
(TIF)

S9 Fig Connections of the vascular system of lateral buds and adventitious roots to the central vascular system of the stem’s axis. A) Longitudinal section of *Tradescantia fluminensis* showing the connection of the internal vascular system of the lateral bud (arrows) to the IVP. B) 3D reconstruction through automatic segmentation of the nodal region of *Dichorisandra thyrsiflora* showing, in transverse plane, the connection of the lateral bud’s vascular system to the IVP. C) Whole-mount diaphanization of the nodal region of *Commelina benghalensis* showing the lateral bud’s vascular system connecting to the vascular system of the stem’s axis. D) Meristematic activity of the adventitious root stained with DAPI, showing the development of the connections with the adjacent peripheral bundles (arrow).
(TIF)
