# Supplementary figures and images for: A new interpretation on vascular architecture of the cauline system in Commelinaceae (Commelinales)
Source: PLoS One. 2019 Jun 20;14(6):e0218383. doi: 10.1371/journal.pone.0218383 (PMC6586312; doi:10.1371/journal.pone.0218383)

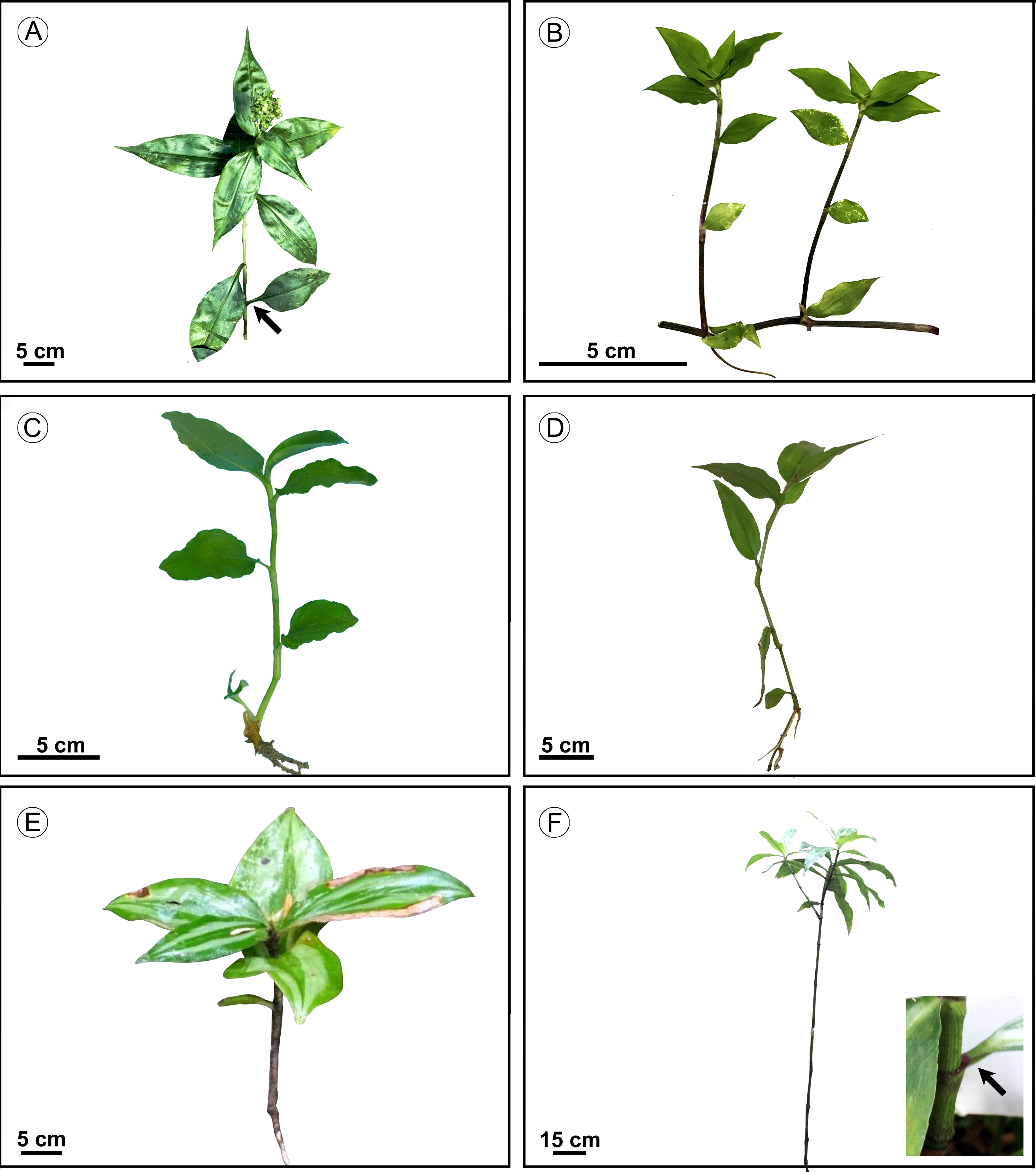

Supplement: S1 Fig — Habit of Aneilema beniniense (P.Beauv.) Kunth (A), Callisia repens (Jacq.) L. (B), Commelina benghalensis L. (C), Commelina rufipes var. glabrata (D.R.Hunt) Faden & D.R.Hunt (D), Dichorisandra radicalis (E), and Dichorisandra thyrsiflora J.C.Mikan (F). Arrow in figure A and F indicate the pseudopetiole. (TIF) [file pone.0218383.s001.tif]

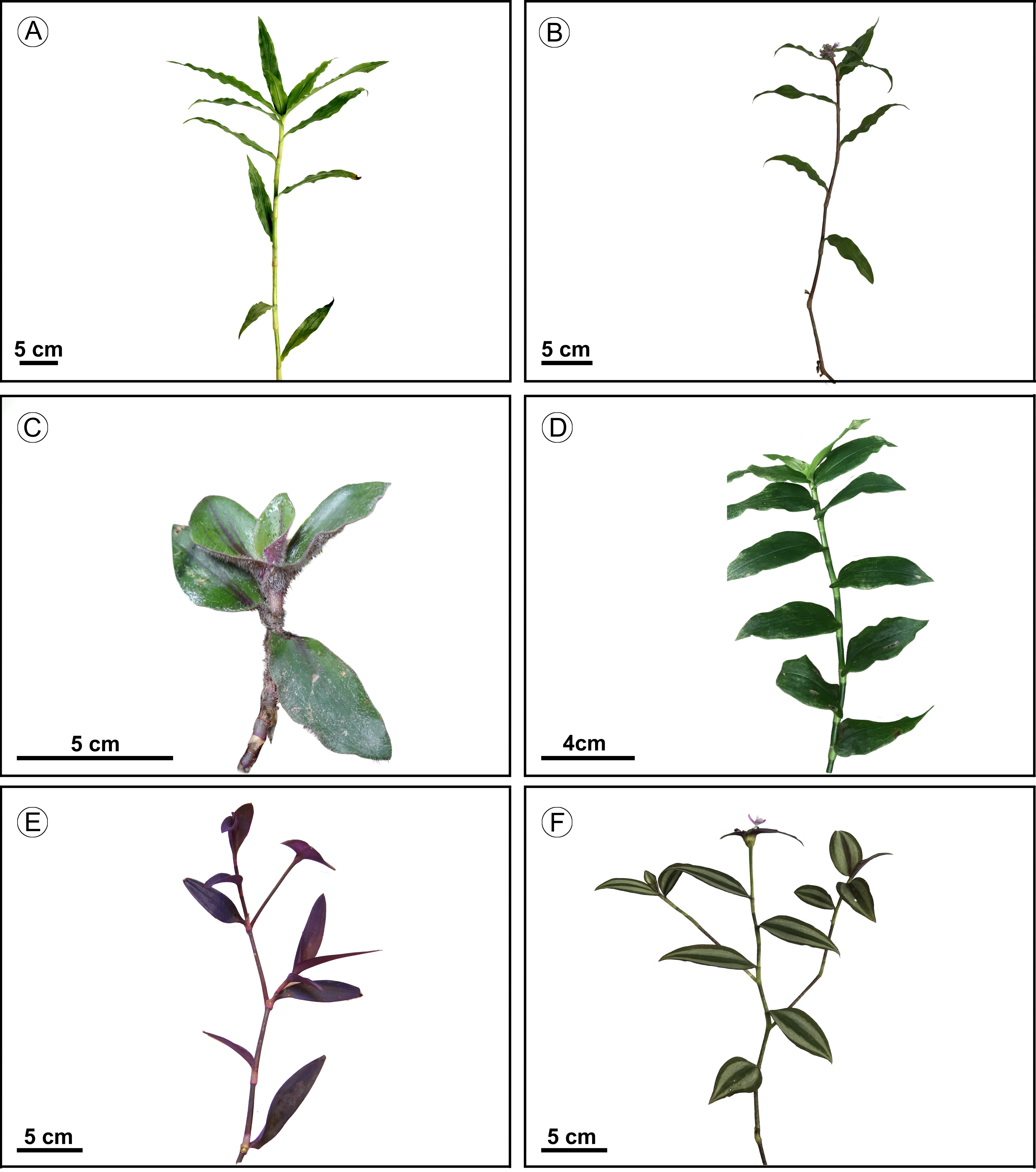

Supplement: S2 Fig — Habit of Floscopa glabrata (Kunth) Hassk. (A), Floscopa aff. glabrata (B), Tradescantia cerinthoides Kunth (C) and Tradescantia fluminensis Vell. (D), Tradescantia pallida (Rose) D.R.Hunt (E), and Tradescantia zebrina Heynh. ex Bosse (F). Arrow in figure B indicating pseudopetiole. (TIF) [file pone.0218383.s002.tif]

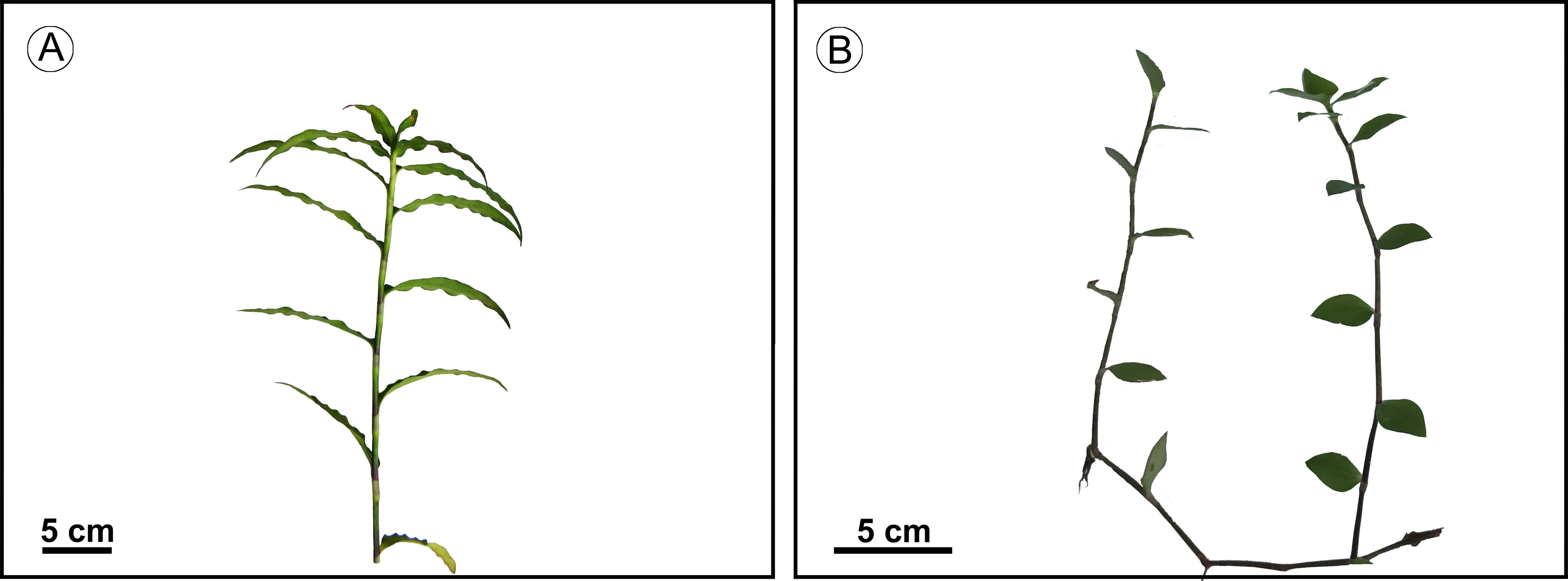

Supplement: S3 Fig — Habit of Tripogandra diuretica (Mart.) Handlos (A) and Tripogandra warmingiana (Seub.) Handlos (B). (TIF) [file pone.0218383.s003.tif]

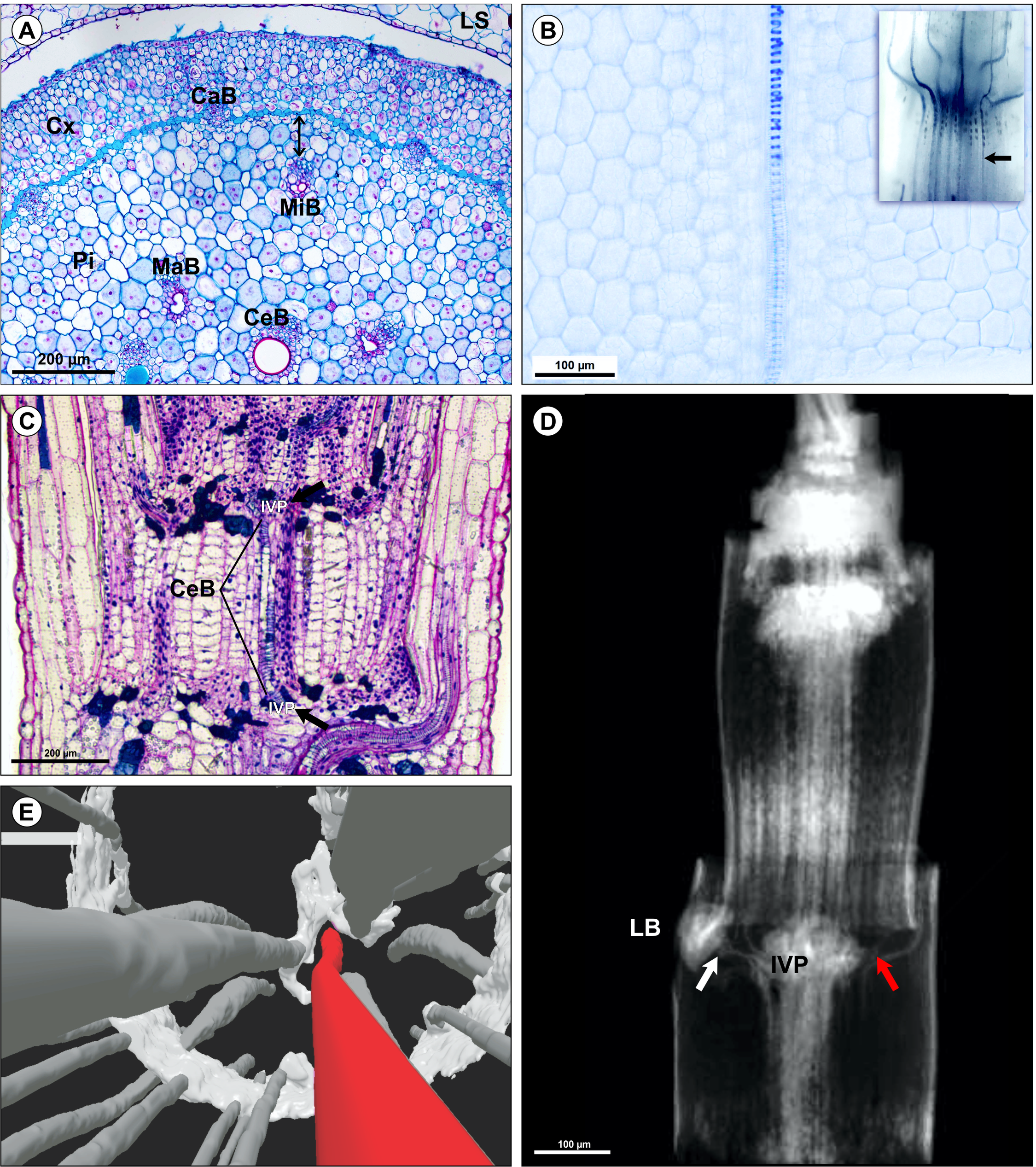

Supplement: S4 Fig — A) Transverse section of Commelina benghalensis showing the position of the minor vascular bundle, slightly away from the pericycle. B) Whole-mount diaphanization showing the late differentiation of a vascular bundle at the base of the internode, intercalary meristem region. C) Longitudinal section of Tripogandra sp. showing the continuity of a central bundle between two IVPs (black arrows). D) μCT analysis by contrast attenuation of the shoot apex of Tradescantia zebrina, contrasted with uranyl acetate. White arrow indicates connection of the internal vascular system of the lateral bud (LB) to the IVP. Red arrow indicates a leaf trace. E) 3D reconstruction by means of manual segmentation of the vascular system showing a major bundle (red) passing through the IVP without connections. (TIF) [file pone.0218383.s004.tif]

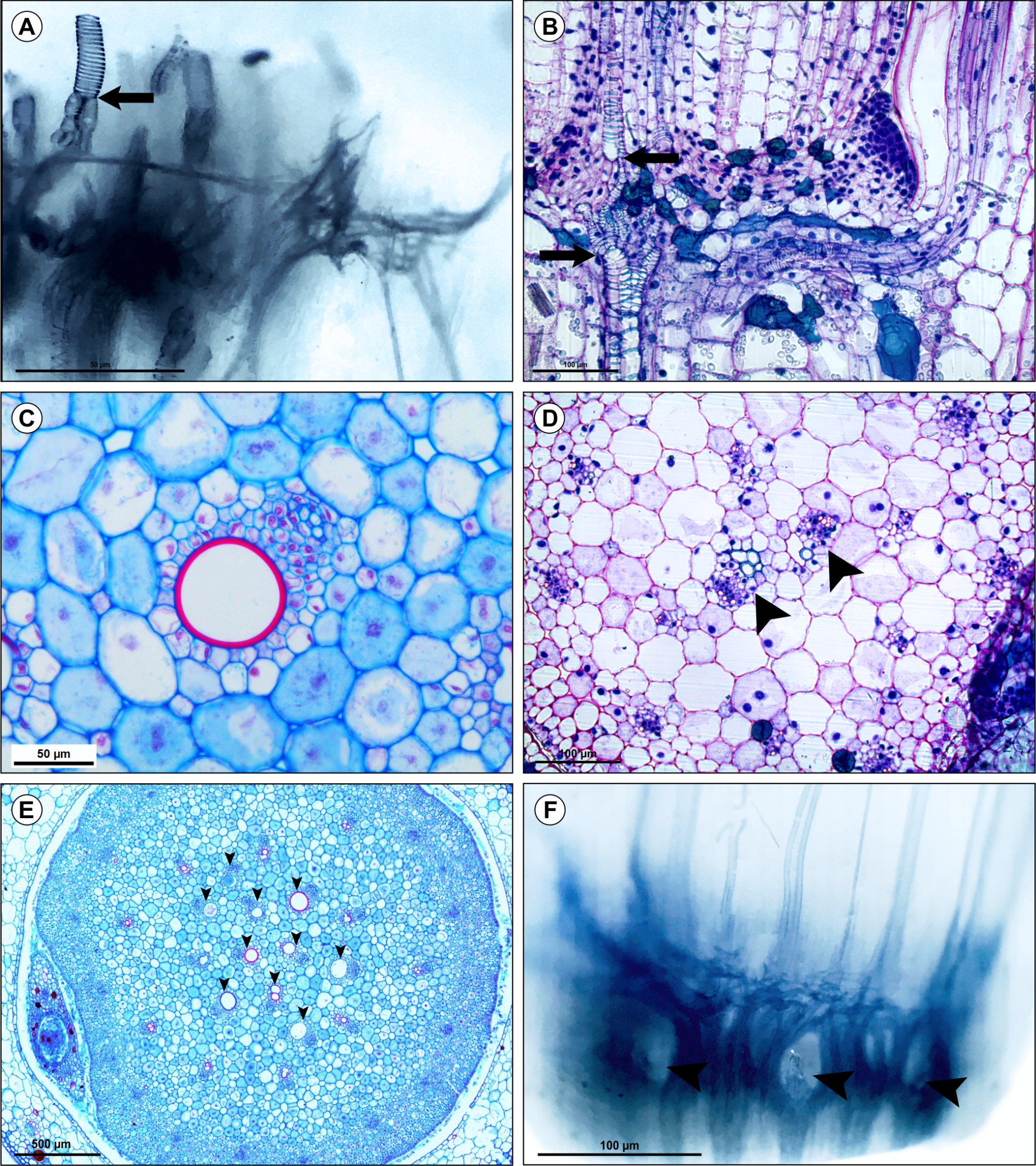

Supplement: S5 Fig — A) Whole-mount diaphanization of the nodal region of Commelina benghalensis indicating a terminal vessel element of the central bundle connecting to the IVP through tracheids (arrow). B) Longitudinal section of Tripogandra warmingiana in the IVP region showing the connection of major bundles to the IVP (arrows). C) Transverse section of the internode of C. benghalensis showing a central bundle only with metaxylem. D) Transverse section of T. warmingiana showing only two central bundles (arrowheads). E) Transverse section of the internode of C. benghalensis with ten central bundles (arrowheads). F) Whole-mount diaphanization of the nodal region of C. benghalensis indicating leaf gaps (arrowheads). (TIF) [file pone.0218383.s005.tif]

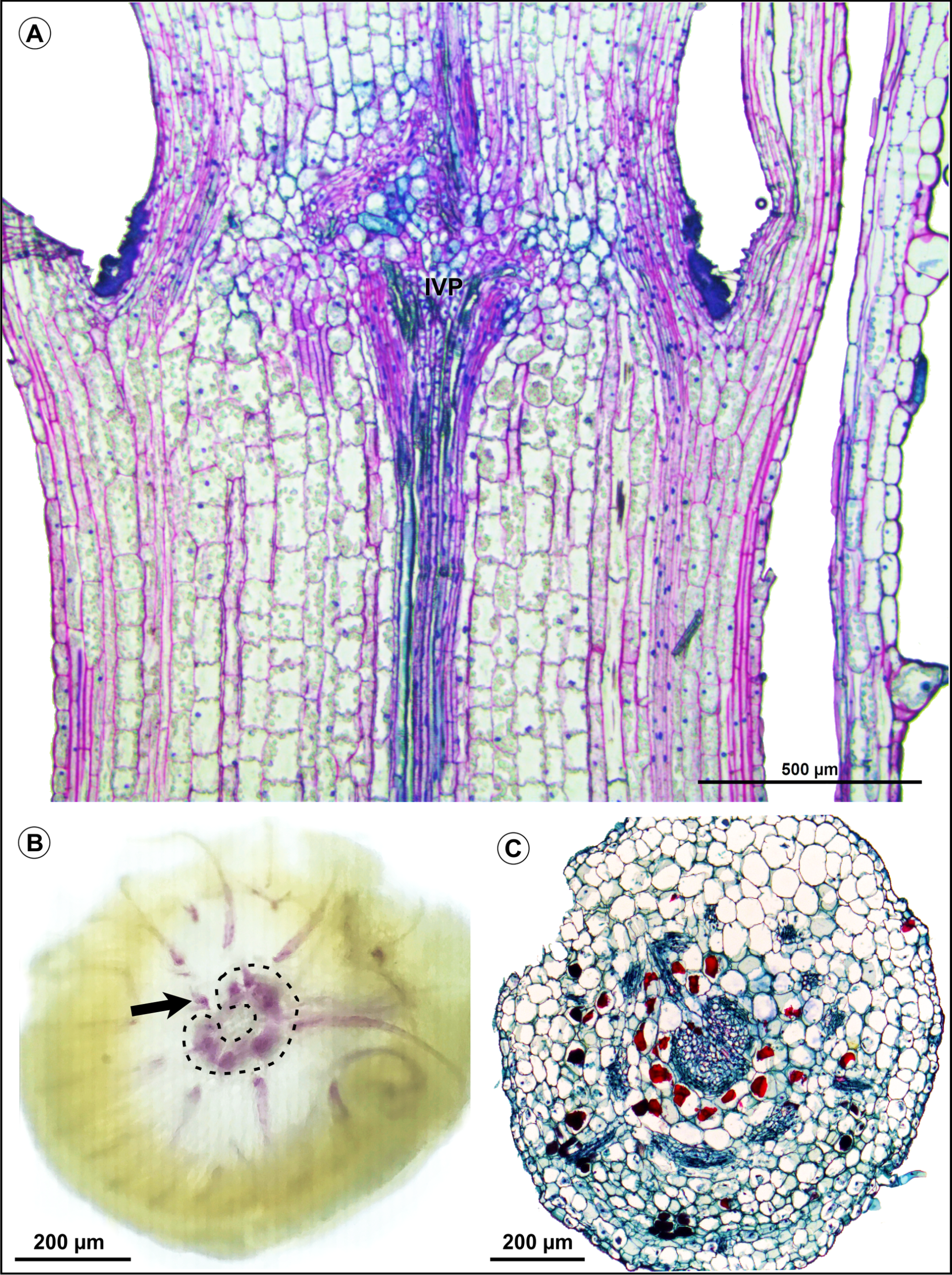

Supplement: S6 Fig — A) Longitudinal section of Dichorisandra radicalis showing the median region of the IVP. B) Diaphanization of the nodal region of Tradescantia zebrina showing the concentric configuration of bundles in the IVP without connection to the EVP. Arrow indicates the major bundle that crosses the node without connection to the IVP. C) Transverse section of Callisia repens with concentric configuration of bundles in the IVP without connection to the EVP. (TIF) [file pone.0218383.s006.tif]

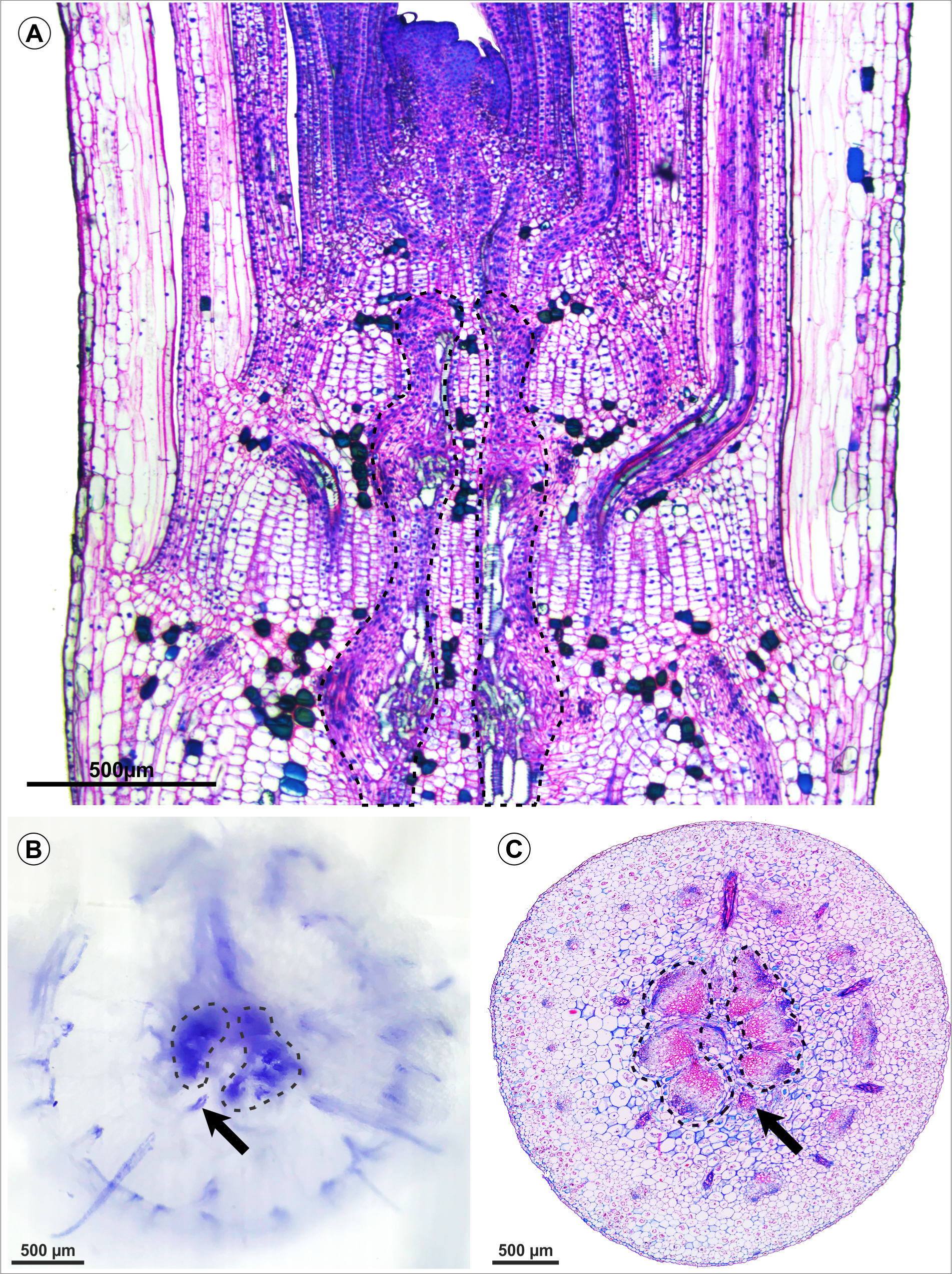

Supplement: S7 Fig — A) Longitudinal section of Tripogandra diuretica showing the median region of the IVP. B) Diaphanization of the nodal region of T. diuretica showing the concentric configuration of bundles in the IVP forming two groups of bundles. C) Transverse section of T. diuretica in the nodal region showing the major bundle that crosses the node without connection to the IVP (arrow). (TIF) [file pone.0218383.s007.tif]

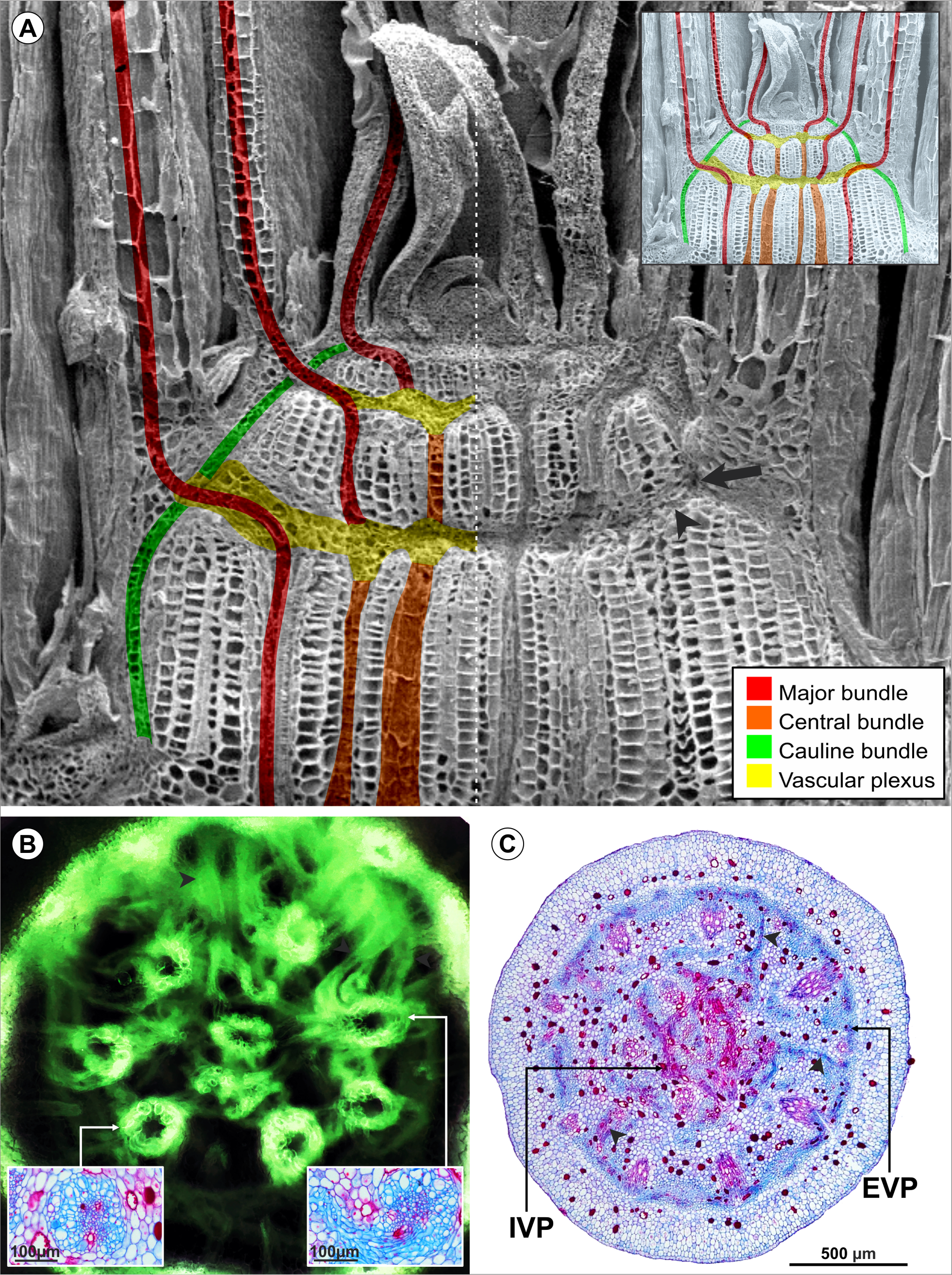

Supplement: S8 Fig — A) Longitudinal section of Commelina benghalensis showing the median region of the IVP. During the assembly process of the shoot apex samples in stubs for scanning electron microscopy, the parenchyma cells absorbed moisture faster than vascular tissue, causing a depression that marked the vascular system throughout the sample. Vascular bundles and plexus of the left meridian were digitally colored. Arrow indicates EVP. B) Diaphanization of the nodal region of C. benghalensis showing the concentric configuration of bundles in the IVP forming two groups of bundles. Arrow indicates the major bundle that crosses the node without connection to the IVP. C) Transverse section of C. benghalensis in the nodal region, showing connections between the IVP and the EVP (arrowheads). (TIF) [file pone.0218383.s008.tif]

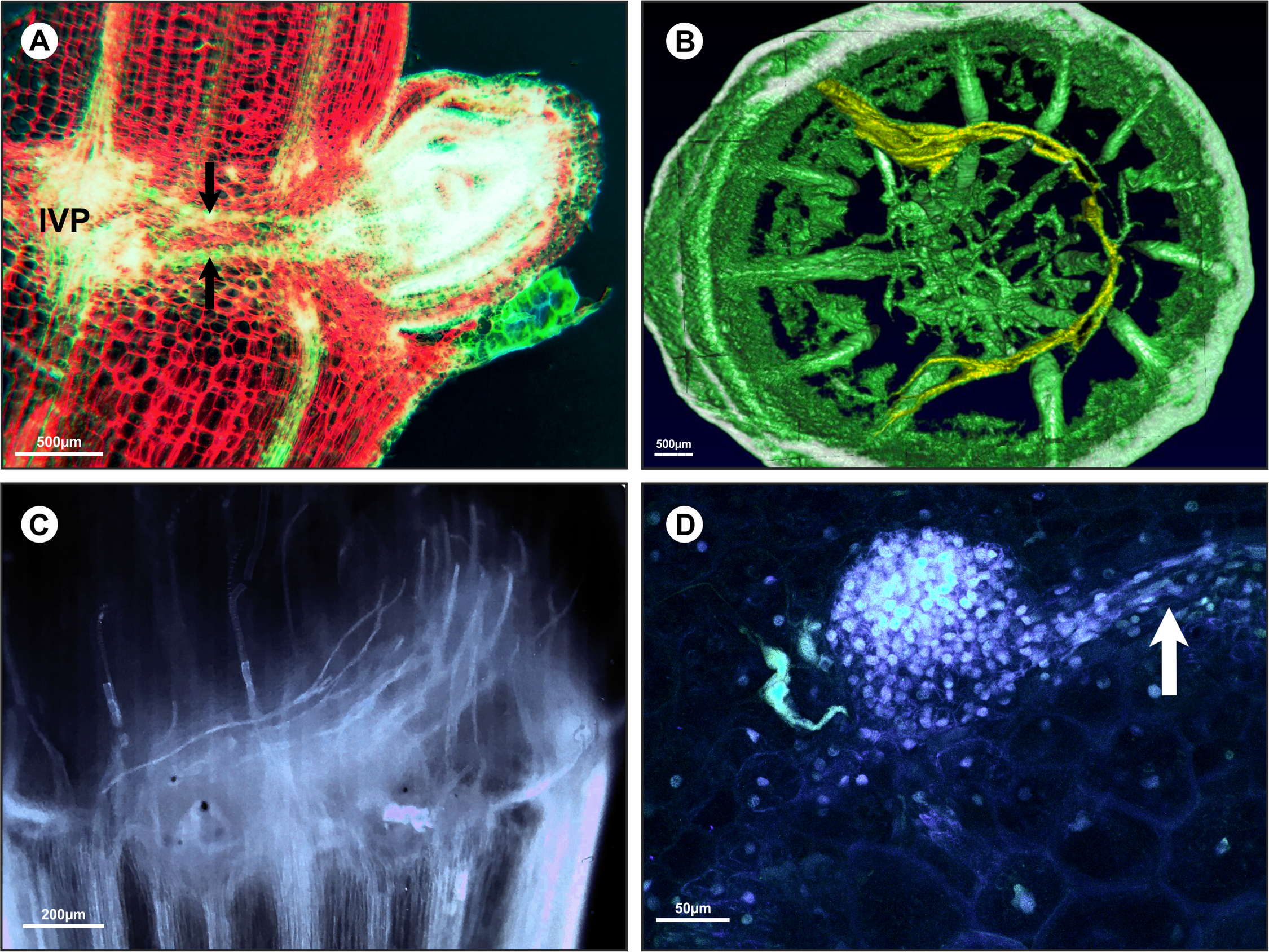

Supplement: S9 Fig — A) Longitudinal section of Tradescantia fluminensis showing the connection of the internal vascular system of the lateral bud (arrows) to the IVP. B) 3D reconstruction through automatic segmentation of the nodal region of Dichorisandra thyrsiflora showing, in transverse plane, the connection of the lateral bud’s vascular system to the IVP. C) Whole-mount diaphanization of the nodal region of Commelina benghalensis showing the lateral bud’s vascular system connecting to the vascular system of the stem’s axis. D) Meristematic activity of the adventitious root stained with DAPI, showing the development of the connections with the adjacent peripheral bundles (arrow). (TIF) [file pone.0218383.s009.tif]
